# Proceedings of Chemistry, Pharmacology, Pharmacokinetics and Synthesis of Biflavonoids

**DOI:** 10.3390/molecules26196088

**Published:** 2021-10-08

**Authors:** Xinqian He, Fan Yang, Xin’an Huang

**Affiliations:** 1 Artemisinin Research Center, Guangzhou University of Chinese Medicine, Guangzhou 510000, China; charilce@foxmail.com (X.H.); 18810959065@163.com (F.Y.); 2 The First Affiliated Hospital of Guangzhou University of Chinese Medicine, Guangzhou University of Chinese Medicine, Guangzhou 510000, China

**Keywords:** biflavonoids, chemistry, pharmacology, pharmacokinetics, synthesis

## Abstract

Biflavonoids, composed of two monoflavonoid residues, occur naturally in angiosperms, bryophytes, ferns, and gymnosperms. More than 592 biflavonoids have been structurally elucidated, and they can be classified into two groups of C-C and C-linear fragments-C, based on whether the linker between the two residues contains an atom. As the linker can be established on two arbitrary rings from different residues, the C-C type contains various subtypes, as does the C-linear fragment-C type. Biflavonoids have a wide range of pharmacological activities, including anti-inflammatory, antioxidant, antibacterial, antiviral, antidiabetic, antitumor, and cytotoxic properties, and they can be applied in Alzheimer’s disease and Parkinson’s disease. This review mainly summarizes the distribution and chemistry of biflavonoids; additionally, their bioactivities, pharmacokinetics, and synthesis are discussed.

## 1. Introduction

Flavonoids, one of the main classes of secondary metabolites in plants, and have representative scaffolds as flavones, chalcones, isoflavones, aurones, and xanthones. Biflavonoids, as members of the flavonoid family, are comprised of two monoflavonoids by a direct connection, or a linear linker. In 2017, Gontijo et al., summarized 139 biflavonoids and their medical applications [1]. In the same year, Sheng Yu et al. [2] also reviewed the phytochemistry, pharmacology, and pharmaceutics of amentoflavone in biflavonoids, including a comprehensive description and summary of the source and current situation of amentoflavone derivatives. It is known that amentoflavone can be obtained from different parts of 127 plants, 45 kinds of derivatives that belong to the same type of connection with amentoflavone. The pharmacological effects of amentoflavone are summarized, including its anti-inflammatory, antioxidation, antitumor, antiaging, antidiabetes, antiviral, central nervous, cardiovascular system, antifungal, and other pharmacological effects. The amentoflavone family is recorded in detail.

In this report, 592 biflavonoids, as well as their distribution, structural scaffolds, and chemical subtype are reviewed. In addition, the pharmacology and synthesis of biflavonoids are summarized.

## 2. Distribution of Biflavonoids

A total of 592 biflavonoids are widely distributed in angiosperms, ferns, gymnosperms, and bryophytes, but most of them are found in angiosperms, including: Anacardiaceae, Apiaceae, Aristolochiaceae, Asteraceae, Balsaminaceae, Berberidaceae, Caprifoliaceae, Chloranthaceae, Clusiaceae (especially Garcinia), Daphniphyllaceae, Ephedraceae, Ericaceae, Euphorbiaceae, Gentianaceae, Juglandaceae, Lanariaceae, Leguminosae, Liliaceae, Lythraceae, Menispermaceae, Moraceae, Myrtaceae, Ochnaceae, Polygonaceae, Rosaceae, Rubiaceae, Theaceae, Thymelaeaceae, Velloziaceae, and Vitaceae. The vast majority of biflavonoids are come from Clusiaceae, Thymelaeaceae, Ochnaceae, and Selaginellaceae, which account for approximately 50% of the biflavonoids in all families. The standard names of the plant families are from The Plant List (2013), which was published in http://www.theplantlist.org/ (accessed on 21 October 2020).

## 3. The Scaffold of Biflavonoids

**Figure 1:**
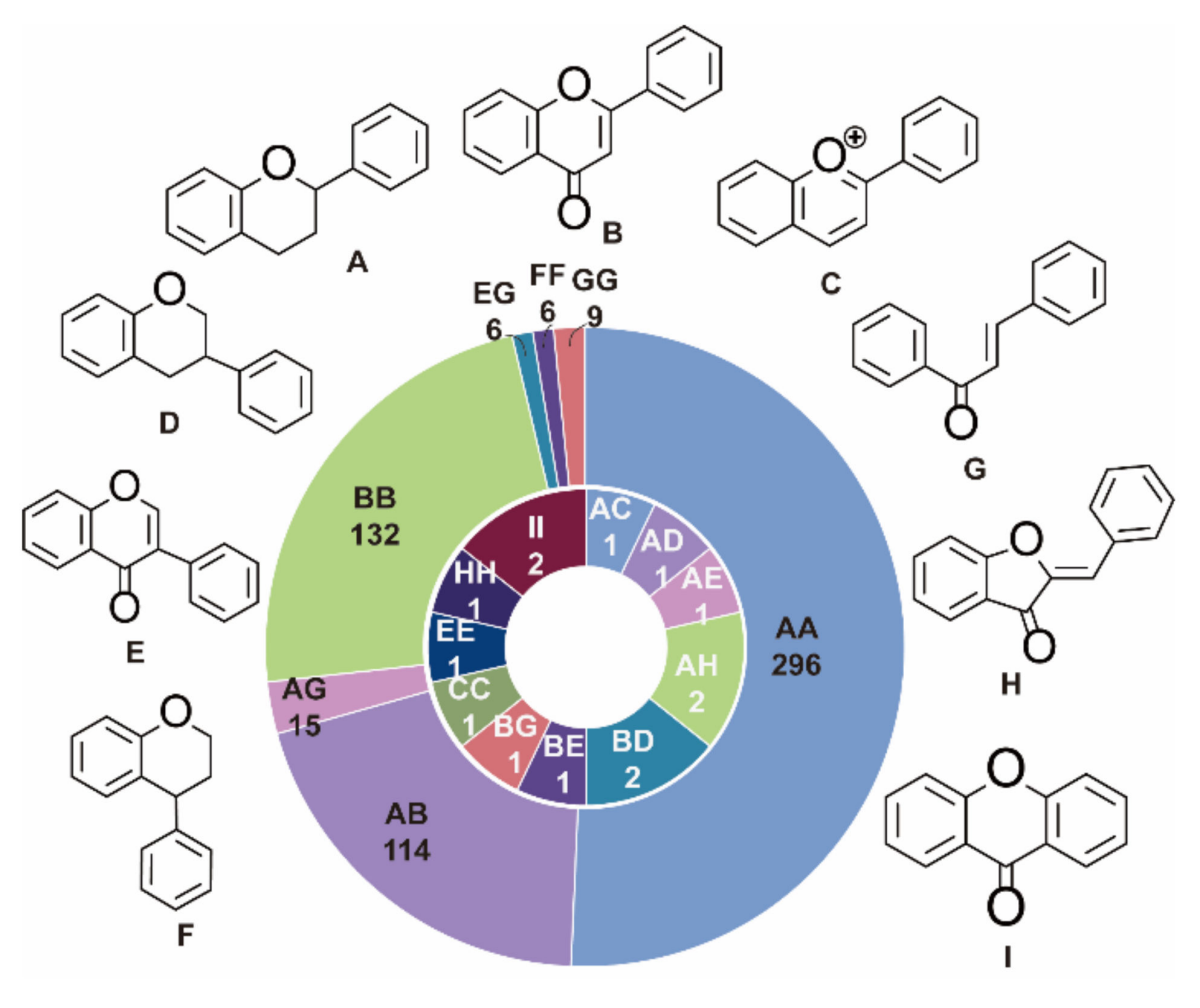
The scaffold of biflavonoids.

In these 592 biflavonoids, according to the C6-C3-C6 combination pattern, flavan (A), flavone (B), anthocyanidin (C), isoflavan (D), isoflavone (E), neoflavan (F), chalcone (G), aurone (H), and xanthone (I) were the main monoflavonoid scaffolds. According to the different monomer combination types, 592 biflavonoids were divided into 17 kinds, including: AA (flavan-flavan), AB (flavan-flavone), AC (flavan-anthocyanidin), AD (flavan-isofalvan), AE (flavan-isoflavone), AG (flavan-chalcone), AH (flavan-aurone), BB (flavone-flavone), BD (flavone-isoflavan), BE (flavone-isoflavone), BG (flavone-chalcone), CC (anthocyanidin-anthocyanidin), EE (isoflavone-isoflavone), EG (isoflavone-chalcone), FF (neoflavan-neoflavan), GG (chalcone-chalcone), HH (aurone-aurone), and II (xanthone-xanthone) (Figure 1). Among them, AA type biflavonoids are abundant in natural plants, and have good development prospects.

## 4. Subtypes of Biflavonoids

### 4.1. C-C Type

According to the connection mode of biflavonoids, they are divided into three major groups.

Group A is about C-C linkages (Table 1, Table 2, Table 3, Table 4, Table 5, Table 6, Table 7 and Table 8); C-C type biflavonoids have a large number, so they can according the positions of their combinations, divide into: 2-3′′, 2′-2′′′, 2′-6′′, 2′-8′′, 3-3′′, 3-3′′′, 3′-3′′′, 3′-4′′′,3′-5′′, 3-6′′, 3′-6′′, 3-7′′, 3′-7′′, 3-8′′, 3′-8′′ , 4-6′′, 4-8′′, 4′-8′′, 5-5′′, 6-6′′, 6-γ, 6-8′′, 7-7′′, and 8-8′′. 

**Figure 2:**
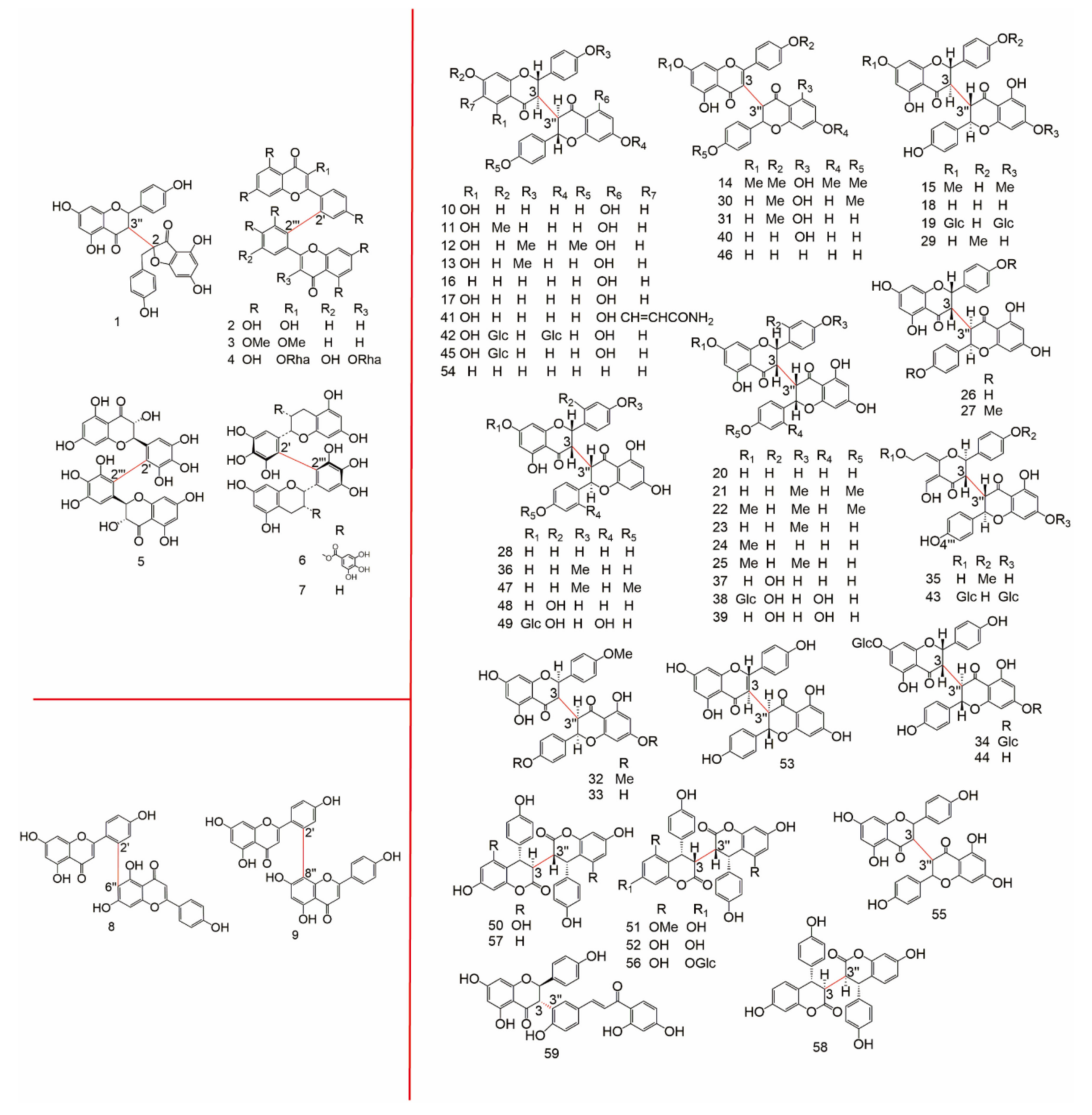
The structure of 2-3′′, 2′-2′′′, 2′-6′′, 2′-8′′, and 3-3′′ type.

The detailed data about subtypes, No., monomer types, origin families and references of 2-3′′, 2′-2′′′, 2′-6′′, 2′-8′′, 3-3′′ type biflavonoids were showed in Table 1, the structure of them were drew in Figure 2. 

**Figure 3:**
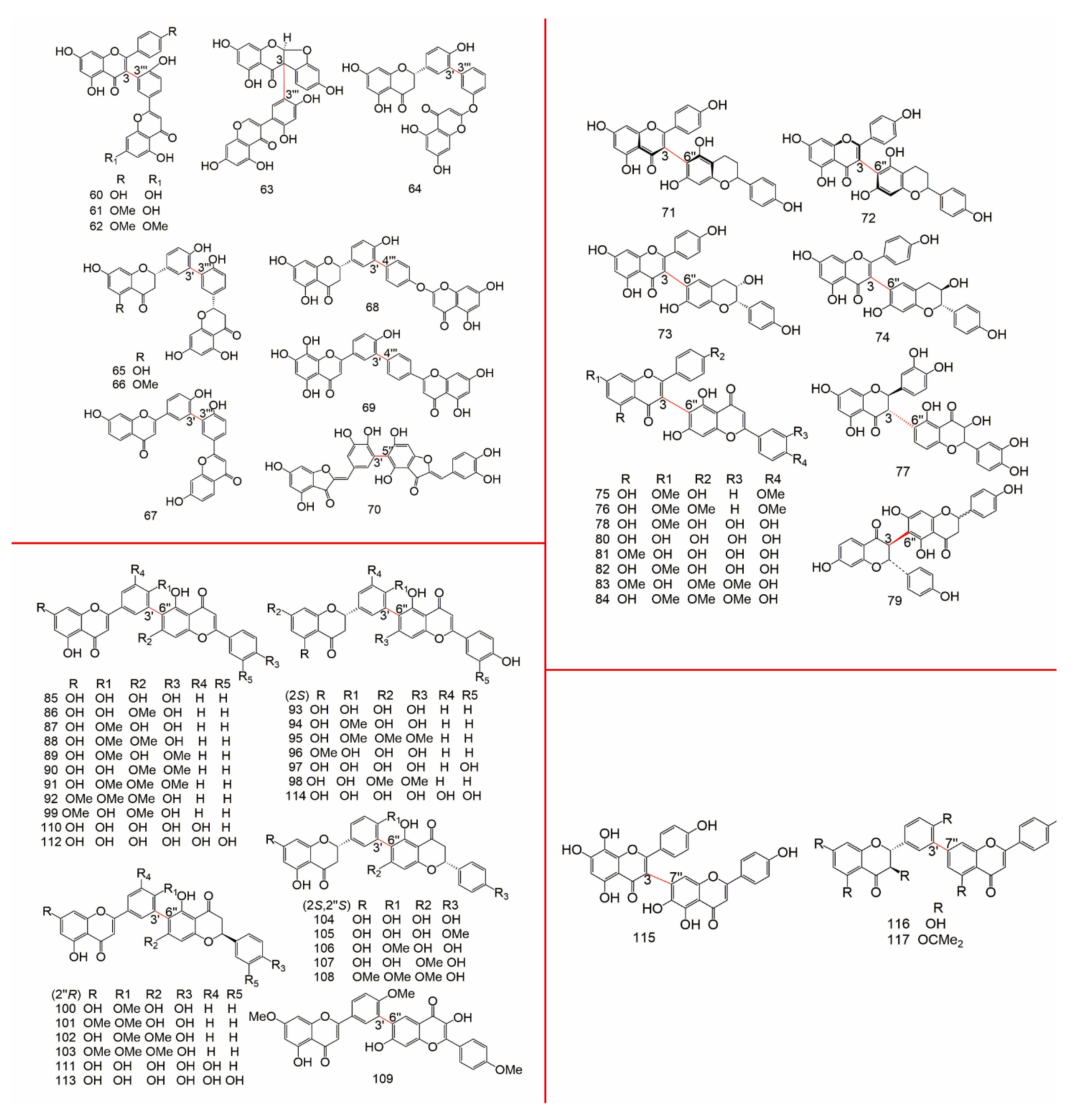
The structure of 3-3′′′, 3′-3′′′, 3′-4′′′,3′-5′′, 3-6′′, 3′-6′′, 3-7′′, and 3′-7′′type.

The detailed data of 3-3′′′, 3′-3′′′, 3′-4′′′,3′-5′′, 3-6′′, 3′-6′′, 3-7′′, 3′-7′′ type biflavonoids were showed in Table 2, the structure of them were drew in Figure 3. 

**Figure 4:**
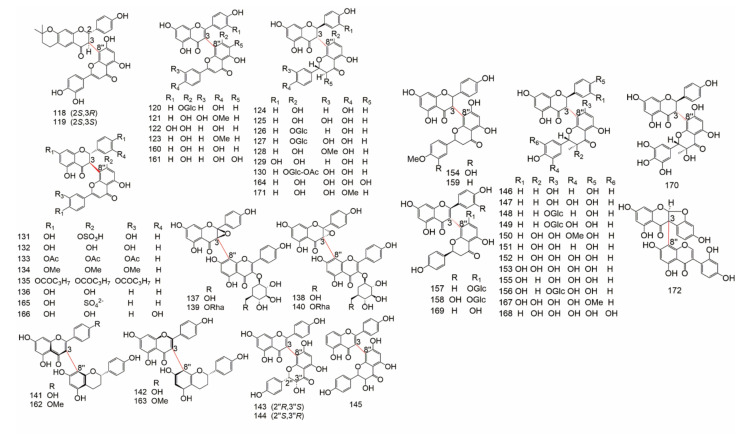
The structure of 3-8′′ type biflavonoids.

The data of 3-8′′ type biflavonoids were showed in Table 3, the structure of them were drew in Figure 4. 

**Figure 5:**
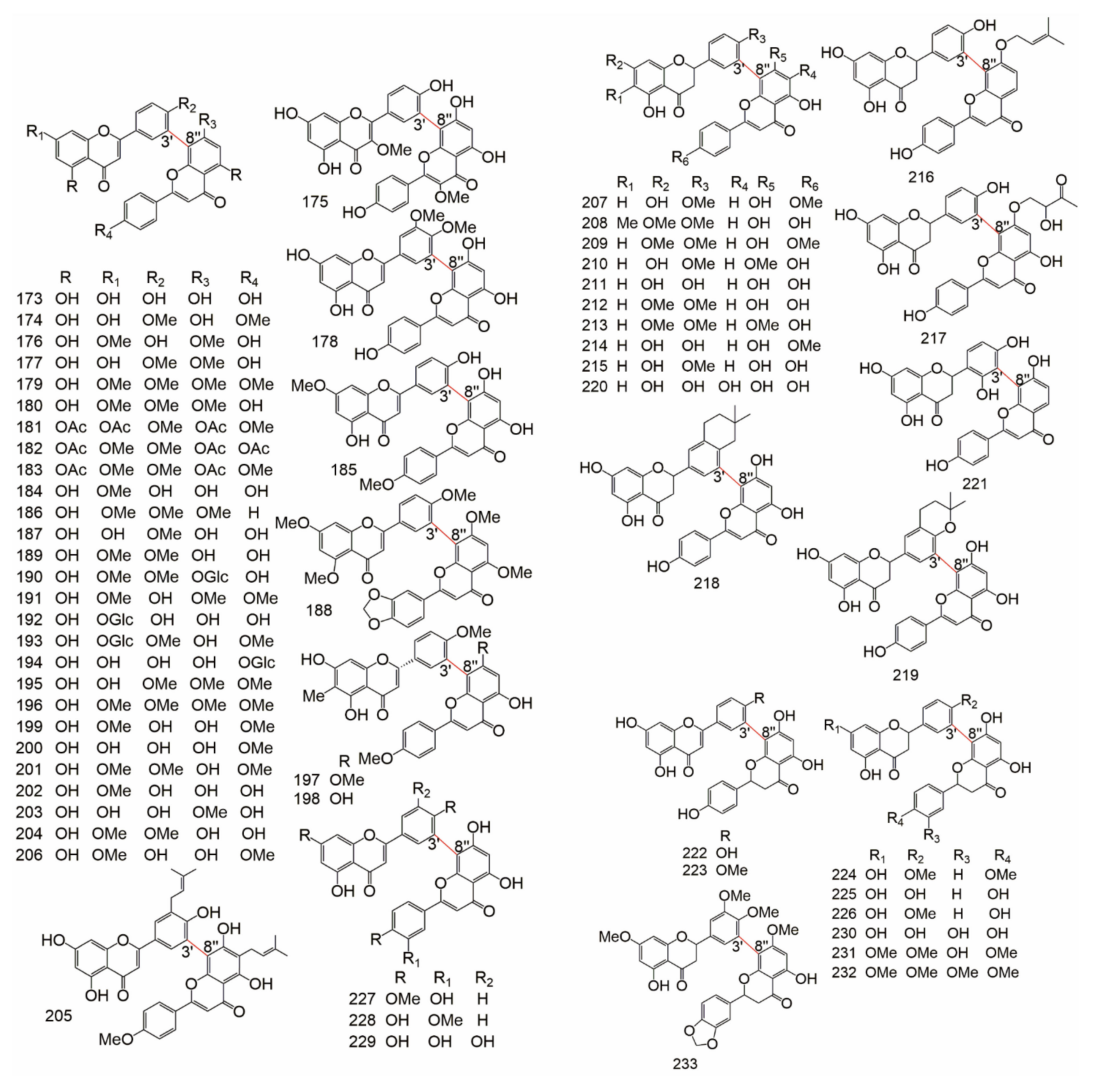
The structure of 3′-8′′ type biflavonoids.

The data of 3′-8′′ type biflavonoids were showed in Table 4, the structure of them were drew in Figure 5. 

**Figure 6:**
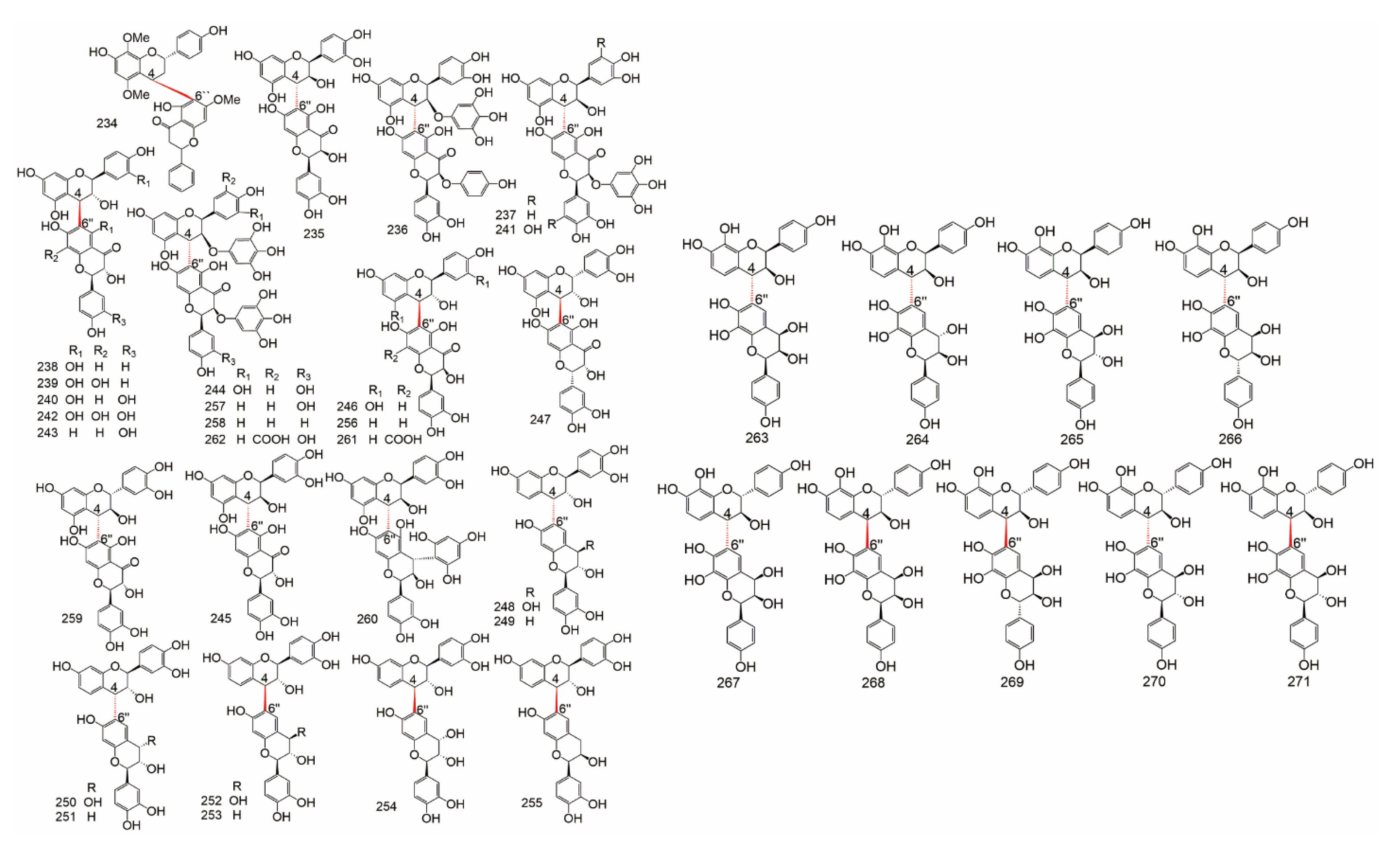
The structure of 4-6′′ type biflavonoids.

The data of 4-6′′ type biflavonoids were showed in Table 5, the structure of them were drew in Figure 6. 

**Figure 7:**
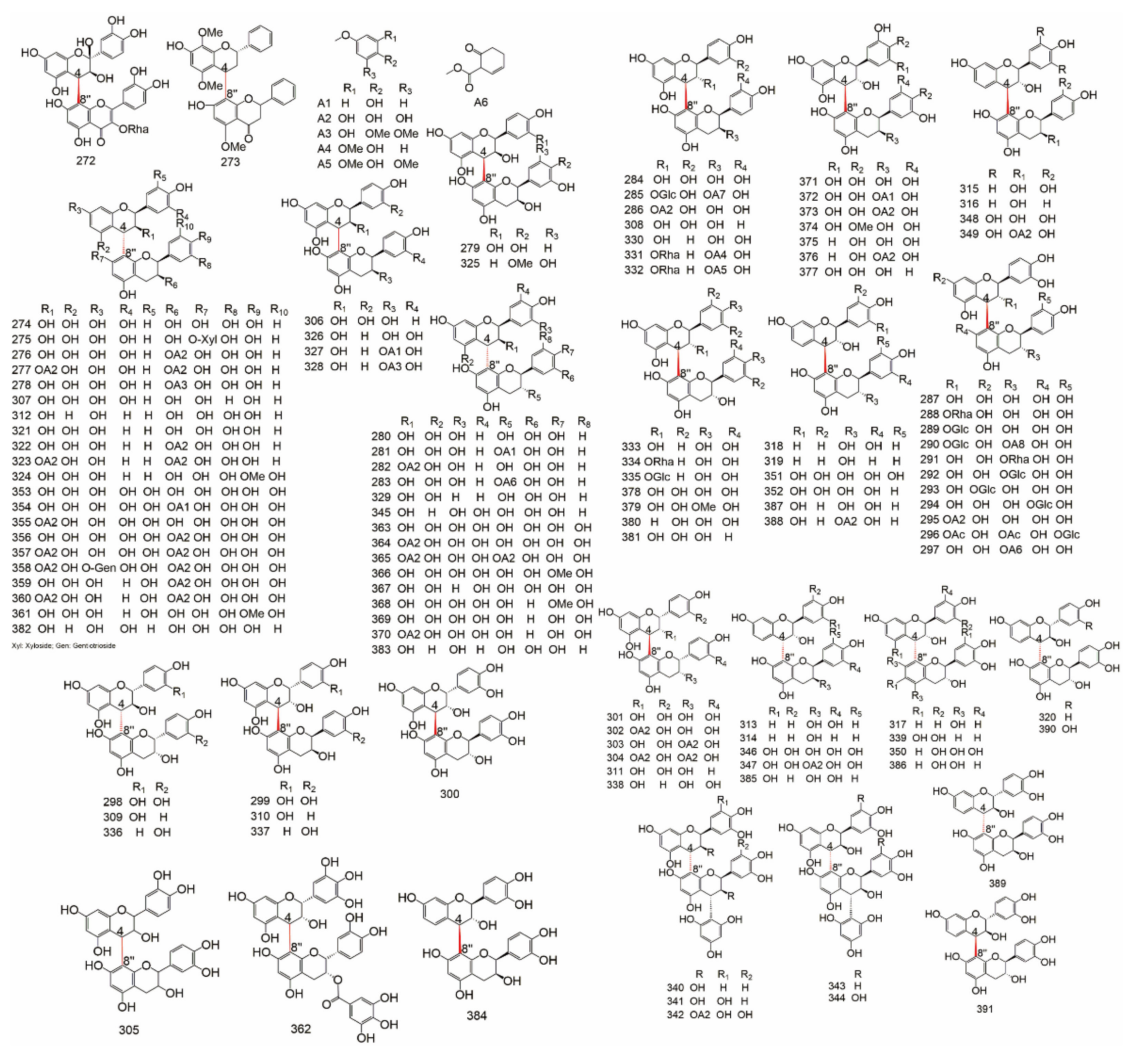
The structure of 4-8′′ type biflavonoids.

The data of 4-8′′ type biflavonoids were showed in Table 6 and Table 7, the structure of them were drew in Figure 7. 

**Figure 8:**
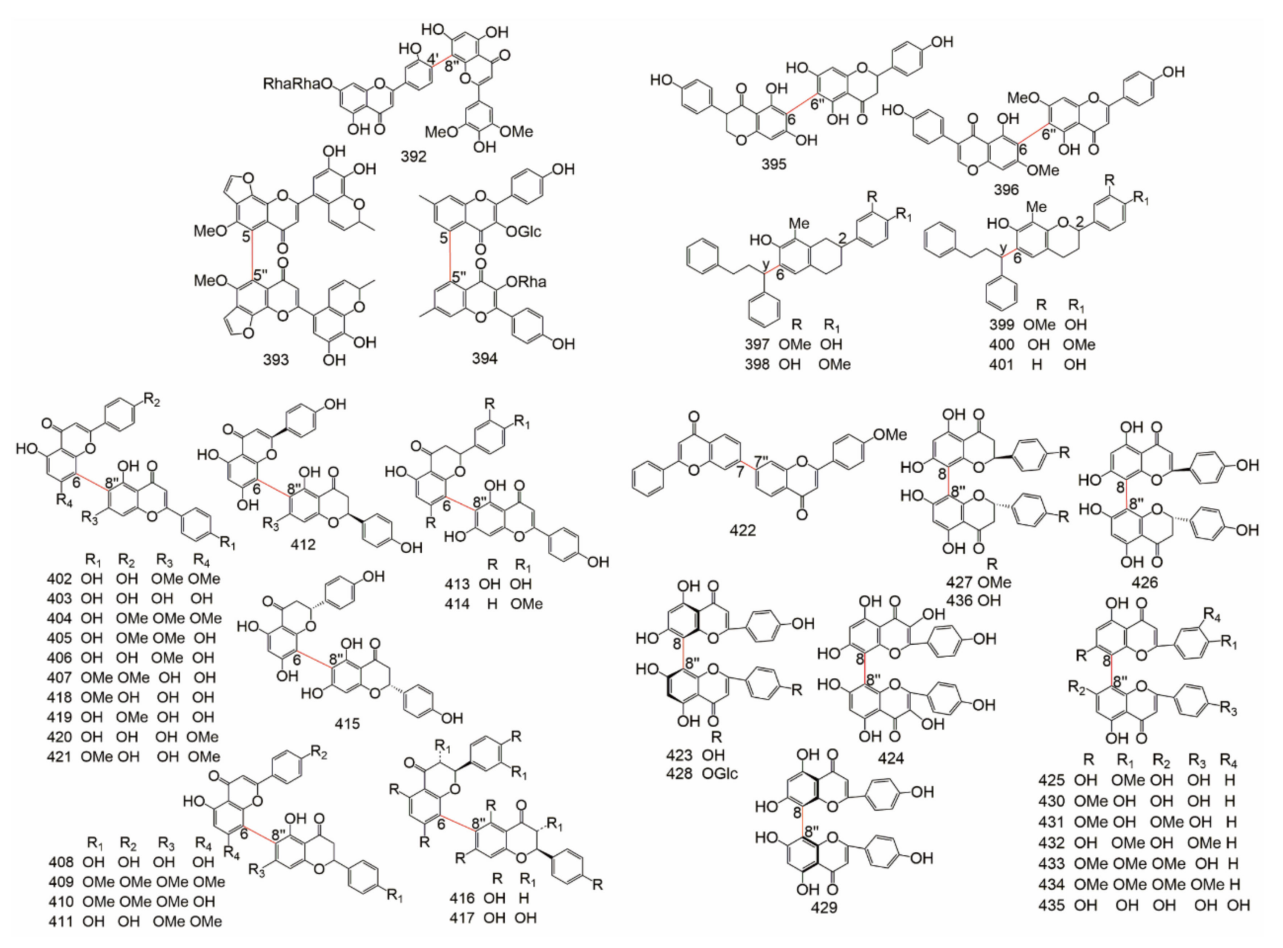
The structure of 4′-8′′, 5-5′′, 6-6′′, 6-γ, 6-8′′, 7-7′′, and 8-8′′ type biflavonoids.

The data of 4′-8′′, 5-5′′, 6-6′′, 6-γ, 6-8′′, 7-7′′, and 8-8′′ type biflavonoids were showed in Table 8, the structure of them were drew in Figure 8.

### 4.2. C-Linear Fragment-C Type

**Figure 9:**
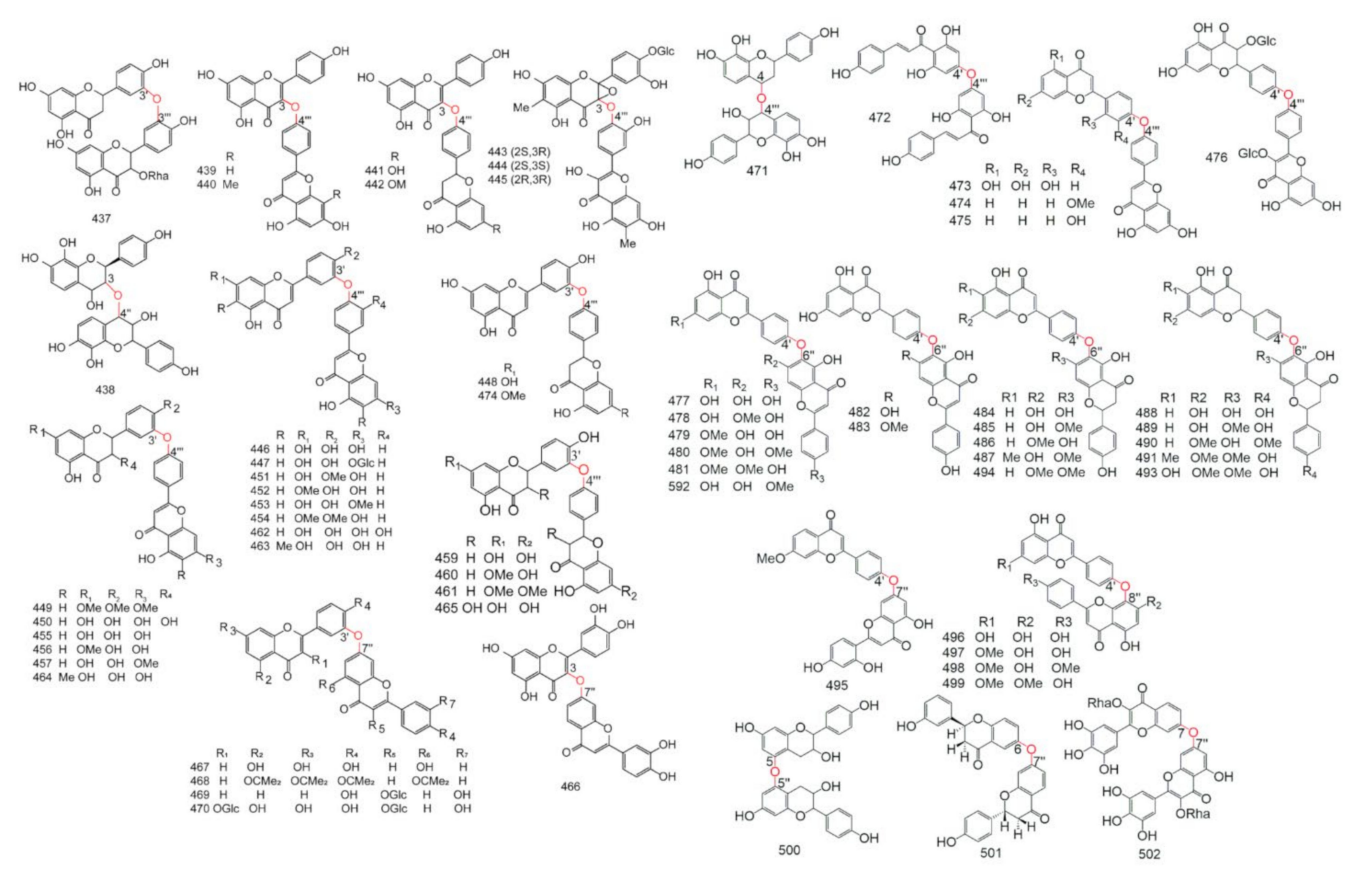
The structure of C-*O*-C type biflavonoids.

**Figure 10:**
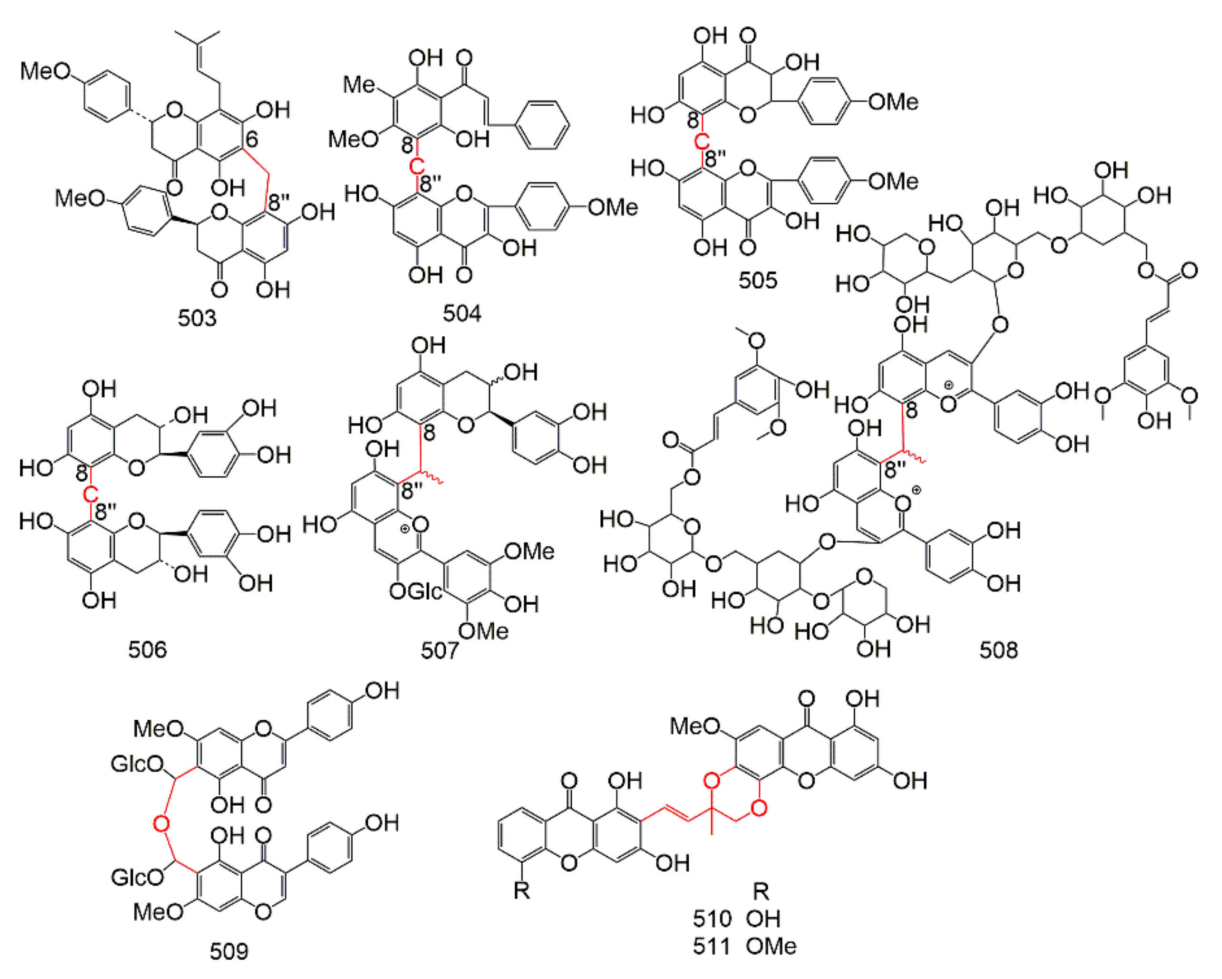
The structure of C-C-C and others type biflavonoids.

Group B (Table 9) is consist of C-*O*-C connections, C-C-C connections and other linear fragment connections, including: 3′-*O*-3′′′, 3-*O*-4′′, 3-*O*-4′′′, 3′-*O*-4′′′, 3-*O*-7′′, 3′-*O*-7′′, 4-*O*-4′′, 4′-*O*-4′′′, 4′-*O*-6′′, 4′-*O*-7′′, 4′-*O*-8′′, 5-*O*-5′′, 6-*O*-7′′, 7-*O*-7′′, 6-C-8′′, and 8-C-8′′. The structure of C-linear fragment-C biflavonoids were showed in Figure 9 and Figure 10.

### 4.3. Complex Biflavonoids

**Figure 11:**
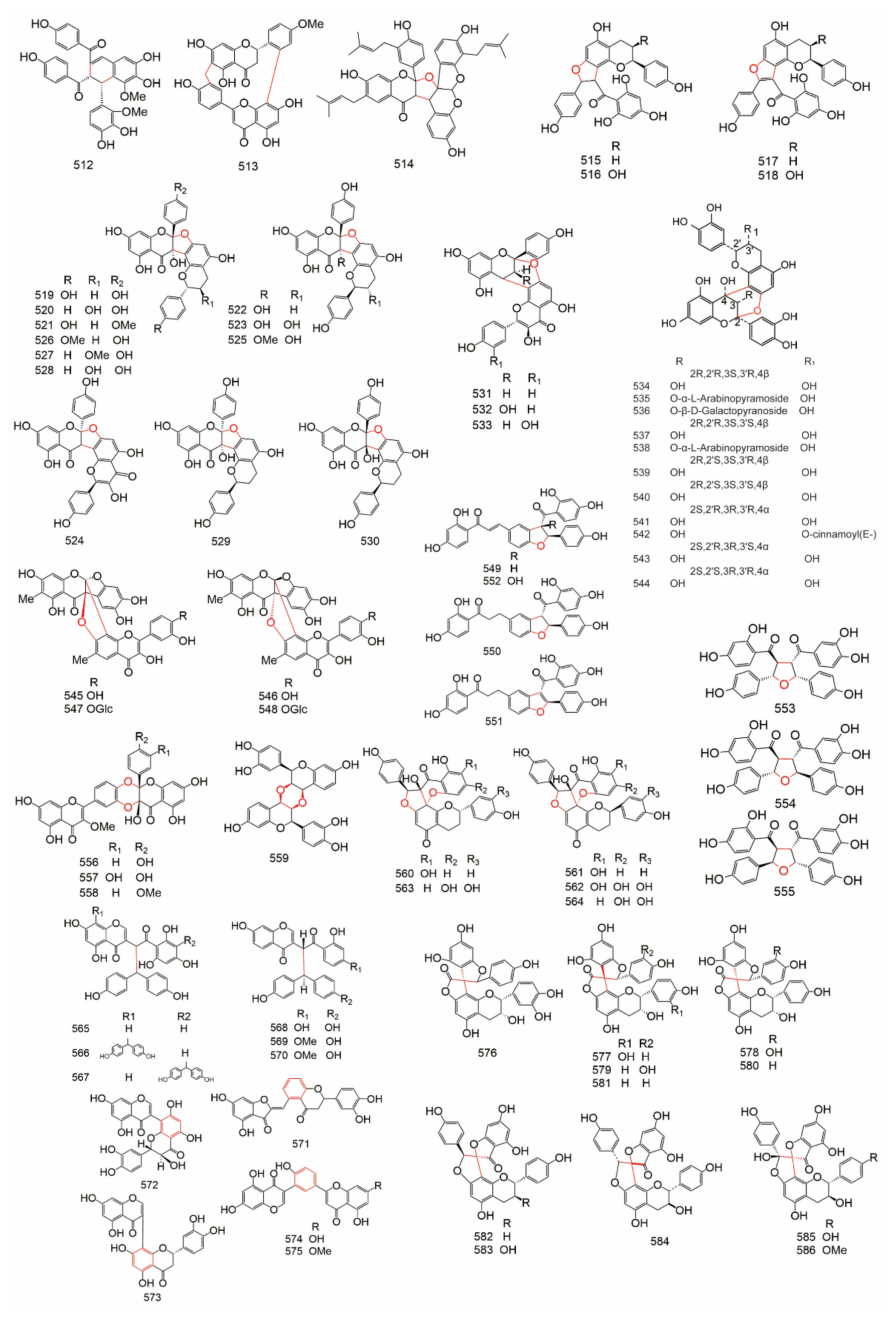
The structure of Complex biflavonoids.

Group C belongs to the complex biflavonoids (Table 10 and Table 11). They include the simple-ring type (C-C & C-C, C-C & C-C-C, C-C & C-*O*-C, C-*O*-C & C-*O*-C), the bicyclic type, the atom-shared type, and spirobiflavonoids. The structure of complex biflavonoids were showed in Figure 11.

## 5. Pharmacology of Biflavonoids

### 5.1. Antioxidant

Andrade et al. [272] conducted an antioxidant test on agathisflavone in 2018. Trolox was used as a control, and agathisflavone was extracted and isolated from the fresh leaves of Caesalpinia pyramidalis Tull. In the experiment of DPPH radical scavenging, it was found that agathisflavone scavenged DPPH free radicals in a concentration-dependent manner; the EC_50_ of agathisflavone was 0.474 mM, and for Trolox it was 0.149 mM, within the 95% confidence interval. The ABTS scavenging assay data found that agathisflavone was EC_50_ = 0.179 mM, while for Trolox, it was EC_50_ = 0.311 mM. In the OH radical scavenging assay, agathisflavone also showed a concentration-dependent hydroxyl radical scavenging ability, while agathisflavone and Trolox both showed a concentration-dependent reduction in the three iron ions to ferrous iron. Through structural analysis of agathisflavone, it was found that the hydroxyl groups at positions 4′,7,7′′,4′′′ in its structure can provide free radical hydrogen to reduce free radicals. In addition, agathisflavone can also inhibit the production of TBARS, and has a significant ability to protect against oxidative damage, indicating that agathisflavone is likely to be a good antioxidant.

The antioxidant effect of Garcinia kola is mainly based on the biflavonoids in the extract. Through the DPPH method and the ATBS method, Lixian et al., studied the antioxidant capacity of garcinianin, kolaflavanone, GB1a, GB2, and panciflavanon. The antioxidant activity of different compounds determined by the DPPH method was garcinianin > panciflavanon > GB2 > kolaflavanone > GB1a, and the antioxidant activity of different compounds determined by the ABTS method was garcinianin > panciflavanon > GB1a > kolaflavanone > GB2. Among them, the antioxidant effect of garcinianin was more obvious [273].

In a study of the antioxidant mechanism of the neuroprotective biflavonoids, hinokiflavone, isocryptomerin, amentoflavone, ginkgetin, amentoflavone, and ginkgetin have good antioxidant capacities, can inhibit the activity of SOD, GR, Gpx, CAT, and other oxidases, reduce the content of GSH, and achieve an antioxidant effect. Ginkgetin can also act on the ERK1/2 target for antioxidants [274]. In 2013, Jia et al. [192] extracted baeckein E from Baeckea frutescens and six other known compounds, and its IC_50_ value ranged from 11.8–16.1 μM in the DPPH free radical scavenging test. Baeckein A and B (IC_50_ = 23.5 μM, IC_50_ = 26.2 μM) showed cytotoxicity and could not be used in H_2_O_2_-induced oxidation experiments. The treatment rates of biflavonoid baeckein E, baeckein C, and baeckein D were 31.8%, 34.8%, and 36.0%, respectively, which were lower than those of nonbiflavonoids (43.0~44.7%).

### 5.2. Anti-Inflammatory Properties

The anti-inflammatory activity of biflavonoids is mainly detected by inhibiting the expression of cyclooxygenase 2 (COX-2) and iNOS. In 2006, Park et al. [275] looked for C-C linked biflavonoids as anti-inflammatory drugs and examined the production of PGE2 and nitric oxide (NO) of synthetic biflavonoids in RAW cells treated with lipopolysaccharide (LPS). The results showed that 3′-6′′, 6-6′′, and 3-4′′′ linked biflavonoids showed resistance to COX-2 -mediated significant inhibition of PGE2 production (IC_50_ = 17.3 μM; IC_50_ = 3.7 μM; IC_50_ = 7.0 μM, respectively). Western blot and reverse transcription-polymerase chain reaction analyses showed that these compounds are not COX-2 downregulation mediated, but are instead COX-2 inhibition mediated. Among them, 6-6′′ biflavonoids have the strongest PGE2 production inhibitory activity. To ensure accuracy, PGE2 and NO tests were performed after LPS pretreatment. The IC_50_ of the 6-6′′ is < 3.0μM, and it can be used as a synthetic leader of new anti-inflammatory agents. However, the biflavonoids 4′-6′′ and 3-4′′′ can have cytotoxic effects on RAW cells.

In 2002, the anti-inflammatory mechanism of amentoflavone as a natural biflavonoid was studied. Banerjee et al. [276] found that amentoflavone can inhibit TNF-α-mediated COX-2 expression through the NF-κB pathway, thereby showing anti-inflammatory effects. In 2019, Li et al. [277] also studied the anti-inflammatory mechanism of the natural biflavonoid ginkgetin, and found that it can produce anti-inflammatory effects through the TLR4/NF-κB signaling pathway and improve ischemia/regeneration perfusion injury.

Jia et al. [255] extracted and separated root products from Baeckea frutescens in 2014 and discovered four new natural biflavonoids of baeckeins F-I. It was found that the four biflavonoids are the cyclic biflavonoids. The conformations of baeckein F, baeckein H (2S, 3S), baeckein G, and baeckein I (2R, 3R) are different, while baeckein H and baeckein I are glycosyl substituted biflavonoids. An anti-inflammatory activity test was performed in the RAW264.7 cell line induced by LPS to produce NO. It was found that the IC50 values of baeckein F, baeckein G, baeckein H, and baeckein I were 54.7 ± 5.26 μM, 25.4 ± 2.78 μM, 43.8 ± 3.30 μM, and 15.2 ± 1.34 μM, respectively, while the IC_50_ of the control indomethacin was 13.8 ± 1.29 μM, and there was no cytotoxicity. Data analysis showed that baeckein H and baeckein I had glycosylated biflavonoids that had more anti-inflammatory activity than the nonglycosylated biflavonoids. The anti-inflammatory activity of baeckein I was not much different from that of indomethacin, and it can be developed as a new anti-inflammatory drug.

There are many mechanisms for the anti-inflammatory activity of biflavonoids. There have been reviews summarizing the anti-inflammatory targets of natural biflavonoids including: ICAM-1, PPAR-γ, COX-2, NF-κB, iNOS, ERK1/2, MMP-9, TIMP-1, and PI3K/Akt, etc. [278]. These are all targets of conventional anti-inflammatory pathways. In addition, predictive pathways such as arachidonic acid metabolism are also new anti-inflammatory mechanisms of biflavonoids.

### 5.3. Antiviral Activities

To find new molecules against dengue fever virus (DV), Coulerie et al. [279] extracted four biflavonoids from the ethyl acetate extract of *Dacrydium balansae*, including amentoflavone, podovarpusflavone A, isoginkgetin, and hinokiflavone, and found that the biflavonoid compounds were the strongest inhibitors of the full activity of DV-NS5 RDRP and DV-NS5, with IC_50_s lower than 3.1 and 5.3 μM. The IC_50_ values were as follows: hinokiflavone (IC_50_ = 0.26 µM) > podovarpusflavone A (IC_50_ = 0.75 µM) > amentoflavone (IC_50_ = 1.40 µM) > isoginkgetin (IC_50_ = 3.10 µM). Hinokiflavone was the most active biflavonoid with IC_50_ = 0.26μM, but podocarpusflavone A was the strongest non-cytotoxic DV-NS5 inhibitor and could inhibit polymerase activity in the DV replicon, so podocarpusflavone A can be used as a template for the development of drugs against dengue fever virus. In addition, amentoflavone can also be developed as an antiviral drug for herpes simplex virus (HSV-1) [280], and agathisflavone can produce an anti-influenza virus effect [281].

### 5.4. Antibacterial and Antifungal Activities

Although the antibacterial and antifungal effects are different in mechanism, this review describes them to facilitate the summary of biflavonoids. Tang et al. [282] isolated six biflavonoids from the bark of *Ochna macrocalyx*. Dehydroxyhexaspermone C, and hexaspermone C are the C-C linked biflavonoids, and ochnone, cordigol, calodenin B, and 2,3-dihydrocalodenin B are all different from general biflavonoids. Calodenin B and 2,3-dihydrocalodenin B have a certain cytotoxicity, but also show strong antibacterial effects. Compared with the control drug, the antibacterial activities of calodenin B and 2,3-dihydrocalodenin B were more obvious. In addition, fukugiside can inhibit the activity of *Streptococcus pyogenes* [283].

The antifungal activity test mainly uses *Candida albicans* to test the antifungal effect of the biflavonoids. Lee et al. [284] used bis-(1,3-dibutylbarbituric acid) trimethine oxonol [DiBAC4(3)], a traditional membrane potential dye, in a regeneration test with fungal protoplasts to study the mechanism of isocryptomerin by depolarization. In this study, amphotericin B was used as a positive control, and isocryptomerin had an MIC value of 18.11 μM, which showed antifungal activity against human pathogenic fungi (such as *Candida albicans* and *Trypanosoma beige*). The cumulative amount of the DiBAC4(3) in isocryptomerin is small and less than the value of amphotericin B, which proves that it destroys the plasma membrane of Candida albicans and causes cell death. In addition, fungal arthritis, caused by *Candida albicans*, and ochnaflavone can promote the expression of IL-2 and IL-10 through the T cell immune system, and inhibit the expression of inflammatory mediators such as IFN-γ and IL-2, but it does not cause hemolysis, kill redundant macrophages, or improve fungal arthritis [285].

### 5.5. Anti-Diabetic and Anti-Atherosclerosis

A biflavonoid composed of two molecules of kaemferol was isolated from the seeds of *Semecarpus anacardium* and its antihyperglycemic mechanism in diabetic mice induced by a high-fat diet plus streptozotocin, was studied showing it could reduce the content of plasma glucose and increase the level of plasma insulin [286]. At a dose of 80 mg/kg b.wt, the biflavonoid’s effect is basically the same as that of metformin, and when the biflavonoid is combined with metformin, they can significantly increase liver and muscle glycogen content, maintain hemoglobin levels, and restore the glycosynthase and glycogen phosphorylase close to normal levels. The glucose metabolism is also maintained at a normal level, and it can significantly increase enzymatic antioxidants (SOD, CAT, GPx, and GST) and nonenzymatic antioxidants (vitamin C, vitamin E, and GSH) and improve the activity of enzymes, thereby curing hyperglycemia. Liu et al. [287] indicated that biflavonoids (isoginkgetin, bilobetin, ginkgetin, and sciadopitysin), which are extracted from *Ginkgo biloba*, have the potential to become pancreatic lipase inhibitors. Four natural biflavonoids had a strong inhibitory effect on pancreatic lipase, and their residual activities were isoginkgetin = 35.7%, bilobetin = 22.3%, ginkgetin = 41.6%, and sciadopitysin = 58.6%. Through the lipase of a concentration-dependent inhibitor of 4-MUO hydrolysis, each IC_50_ value was isoginkgetin = 2.90 ± 0.98 μM, bilobetin = 3.57 ± 0.53 μM, ginkgetin = 6.90 ± 1.60 μM, and sciadopitysin = 12.78 ± 2.30 μM, showing a degree of medium to strong inhibition. Isoginkgetin can also improve the healing of foot ulcer wounds in diabetic rats [288].

There are many pathological mechanisms of atherosclerosis, but they are related to hypertension, hyperlipidemia, and other mechanisms. Therefore, the treatment of atherosclerosis is basically inseparable from the antioxidant and anti-inflammatory effects [289]. Tabares-Guevara et al. performed oxygen radical absorbance capacity (Orac) and lDl oxidation inhibition assays on three natural biflavonoids: morelloflavone, volkensiflavone, and fukugiside, and found that all of them were effective reactive oxygen scavengers, inhibited the production of reactive oxygen species and the secretion of proinflammatory factors (IL-6, IL-12p70, MCP-1, TNF-α, MIP-1α, and NLRP3, etc.) in macrophages, and they reduced the circulating levels of cholesterol and the lipid peroxidation product propylene glycol, showing the antioxidation, anti-inflammatory, hypolipidemic, and anti-atherosclerotic effects of biflavonoids in the body [290].

### 5.6. Alzheimer’s Disease and Parkinson’s Disease

Alzheimer’s disease in terms of anti-inflammatory, antioxidative stress, and neurodegenerative damage overlaps to a large extent with the treatment pathway of biflavonoids [291] so biflavonoids have great potential in the treatment of Alzheimer’s disease [292]. Moreover, due to the aromatic interaction of biflavonoids, their therapeutic effect is better than that of a flavonoid [293], indicating that biflavonoids can be used as lead compounds for the development of treatments for Alzheimer’s disease. In particular, the amentoflavone type includes amentoflavone (1) and its monomethoxy derivatives. They can inhibit the formation and accumulation of amyloid β, thereby preventing Alzheimer’s disease [294].

Choi et al. used the peptide of Aβ1-42 to inhibit the aggregation of Aβ1-42 in vitro by thioflavin T fluorescence analysis of biflavonoids (amentoflavone, bilobetin, sequoiaflavone, sotetsuflavone, podocarpuflavone, ginkgetin, isoginkgetin, and sciadopitysin), and found that amentoflavone has the strongest comprehensive strength in inhibiting the formation of Aβ1-42 fibers and reducing the formation of Aβ1-42 fibers among the eight biflavonoids, and it has great potential as a lead compound for treating Alzheimer’s disease [295]. CGY-1 [82], GB1, and other gambogic biflavonoids [296] also have the potential to treat Alzheimer’s disease.

Biflavonoids extracted from Impatiens balsamina can prevent the production of NO, have neuroprotective activity, and improve neurodegenerative diseases [61]. Amentoflavone can improve Parkinson’s disease through the PI3K/Akt and ERK signaling pathways [297], while ginkgetin can improve Parkinson’s disease nerve damage through neuroprotection [298].

### 5.7. Cytotoxic Activity and Antitumor Activities

**Figure 12:**
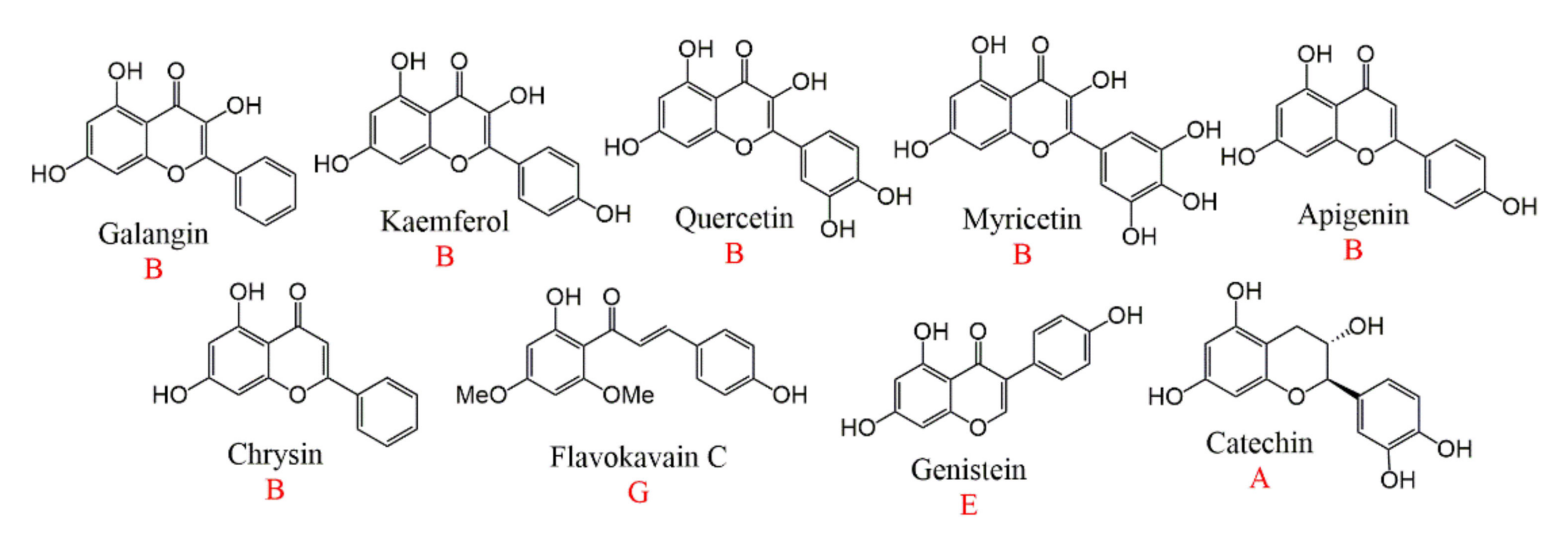
The structure of flavonoids.

The cytotoxicity of flavonoids with different structures is also different. A review had summarized that the flavonoids with flavone(B) units (galangin, kaempferol, quercetin, myricetin, apigenin, and chrysin) had the ability to antihepatoma; the flavonoids with chalcone(G) units (flavokavain C) could cause hepatic failure; the flavonoid with isoflavone(E) units (genistein) had an antiestrogen, increasing the risk of breast cancer and the flavonoids with flavan(A) units (catechin) had no effect on tumor cells, but had the hemolytic anemia thrombocytopenia [299]. Biflavonoids are composed of two flavone monomers, so the toxicity study of flavonoids is also helpful to the toxicity activity of biflavonoids. The structure of these flavonoids are shown in Figure 12.

For the toxicity of biflavonoids, a study found that amentoflavone, sciadopitysin, ginkgetin, isoginketin, and bilobetin extracted from ginkgo can reduce the cell viability of human renal tubular epithelial cells (HK-2 cells) in a dose-dependent manner. Ginkgetin, isoginkgetin, and bilobetin showed the cell viability of HK-2 cells were less than 50% at 10 and 100 μg/mL. At the dose of 100 μg/mL, amentoflavone, ginkgetin, isoginkgetin, and bilobetin injured the human normal hepatocytes (L-02 cells), moreover, the cell viability of isoginkgetin and bilobetin were less than 50%. After HE staining of mouse liver sections, it was found that bilobetin and ginkgetin were more toxic to hepatocytes. In renal tissue, these five biflavonoids caused acute renal injury, and renal interstitial hemorrhage was a common pathological phenomenon [300]. Therefore, *Ginkgo biloba* extract preparation should pay attention to its hepatorenal toxicity. A study found that hinokiflavone, as the cytotoxic principle, its ED_50_ value was 2.0 μg/mL in KB cells. It was proven that the ether bond between the two flavonoid monomers had a significant cytotoxicity. However, other biflavonoids with C-C linkages, being hexamethyl ethers of ring C/A-linked dimers between two flavonoid units, also showing the cytotoxic activity (the ED_50_ value was 3.0~4.0 μg/mL) [301]. A non-clinical toxicological study in 2019 revealed that there were no reported fatalities after agethisflavone acted on the female mice, and it has an LD_50_ larger than 2000 mg/kg [302].

Adem et al. [10] used the caspase-Glo assay to test the cytotoxicity of three biflavonoids (chamaejasmin, 7,7′′-di-*O*-methylchamaejasmin, and campylospermone A) and other compounds. The cell cycle, apoptosis, mitochondrial membrane sites, and reactive oxygen species were analyzed by flow cytometry. The model cells were CCRF-CEM leukemia cells and CEM/ADR5000 cells and seven other cancer cells including U87MG. = EGFR glioblastoma, HepG2 liver cancer cells, U87MG. = EGFR cells, MDA-MB-231/BCRP breast cancer cells, MDA-MB-231 cells, and HepG2 cells. The IC_50_ values of chamaejasmin in CCRF-CEM cells and CEM/SDR5000 cells were both greater than 61 μM, and the IC_50_ value of campylospermone A to CEM/ADR5000 cells was also greater than 61 μM. Therefore, chamaejasmin and campylospermone A were considered to be less cytotoxic. However, 7, 7′′-di-*O*-methylchamaejasmin had an IC_50_ = 3.58 ± 0.09 μM for CCRF-CEM cells, and an IC_50_ = 5.69 ± 0.51 μM for CEM/ADR5000 cells, and the IC_50_ values of the other cancer cells were less than 8 μM, indicating that it had greater cytotoxicity, and could inhibit the growth of cancer cells.

Due to the cytotoxicity of biflavonoids, they have great potential in the treatment of cancer. For example, delicaflavone can inhibit the PI3K/Akt/mTOR and Ras/MEK/Erk signaling pathways in rectal cancer cells through the mitochondrial ROS pathway [303], inhibit the MSPK signaling pathway in HeLa cervical cancer cells, and induce cell apoptosis in G2/M phase [304]; hinokiflavone inhibits the induction of apoptosis of the NF-κB signaling pathway in liver cancer cells by activating the mitochondrial ROS/JNK/caspase pathway [305]. However, the spirobiflavonoids of abiesinolA-F extracted from Abies sachalinensis can effectively inhibiting the activation of NOR1, thereby inhibiting the activity of skin cancer [306]. In the literature on the toxicology of biflavonoids, only the toxicological experiments of biflavonoids with Aunits, B units, and spirobiflavones were included. For instance, 6-8″ linkage biflavonoid (agathisflavone) had no cytotoxic activity [302], but 3′-8″ linkage biflavonoids (amentoflavone, ginkgetin, isoginkgetin, and bilobetin) impaired the liver and renal cells [300] and 3-3″ linkage biflavonoids (chamaejasmin, 7,7″-di-O-methylchamaejasmin, and campylospermone A) had the capacity to inhibit the growth of cancer cells [10]. The biflavonoids with the ether bond between two flavonoid monomers (delicaflavone [301,304], hinokiflavone [305], and spirobiflavone [306]) had the ability of anticancer. Additionally, the biflavonoids with the hexamethyl ether substituents could reduce cell activity [301].

### 5.8. Anti-Angiogenesis

Li et al. [307] correlated zebrafish angiogenesis measurement with ultra-performance liquid chromatography-quadrupole-time of flight-mass spectrometry (UPLC-Q-TOF/MS) as the base chemometric analysis to identify the potential antiangiogenic active compounds of *Garcinia xanthochymus*. Preliminary biological activity results showed that amentoflavone can significantly inhibit the growth of subintestinal vessels at 10 and 20 μM, and downregulate the expression of the Angpt2 and Tie2 genes in zebrafish embryos. In addition, the zebrafish model was used to evaluate the structure-activity relationship of seven biflavonoids (volkensiflavone, fukugetin, fukugeside, GB 1a, GB 1a with glycosides, GB 2a and GB 2a with glycosides) isolated from *Garcinia*. Fukugetin, which has anticancer effects, and can effectively inhibit the growth of subintestinal vessels. Both amentoflavone and fukugetin showed antiangiogenic effects on zebrafish for the first time [308].

### 5.9. Other

In addition, the other pharmacological effects of biflavonoids are: morelloflavone has 63% preventive inhibition of PLA2-induced myotoxic activity, its 38% cure rate inhibits myotoxicity, and it can inhibit edema formation and anticoagulation in a concentration-dependent manner, proving that morelloflavone can be developed as an inhibitor of secretory PLA2 such as in snake venom [309]; GB1 can inhibit α-glucosidase (IC_50_ = 0.90 ± 0.01 mM) and aromatase (IC_50_ = 0.28 ± 0.02 mM), and produce anti-plasmodium activity [310]; robustaflavone-4′-dimethyl ether can inhibit the accumulation of inflammatory cells by inhibiting the AKT and APK pathways, improve lung tissue damage, and reduce pulmonary edema [311]; rhusflavone, from Rhus parviflora, has a sedative and hypnotic effect, significantly binds to the GABAA-BZD receptor (IC_50_ = 0.045 mM), and induces sleep [178]; II-3, I-5, II-5, II-7, I-4′,II-4′-hexahydroxy-(I-3,II-8)-flavonylflavanonol, from G arcinia nervosa var. pubescens King can produce 73.9% in 18.2 μg/mL Platelet-Activating-Factor Inhibition (IC_50_ = 20.4 μM) [312]; and GB-2a-II-4′-OMe has a certain analgesic effect on the pain sensation induced by Marfrine, and its mechanism from analgesic effect is different of morphine [64]; amentoflavone can reduce the influence of gamma rays [313], and six biflavonoids of *Araucaria angustifolia* can improve DNA damage caused by ultraviolet radiation, including: amentoflavone, mono-*O*-methylamentoflavone, di-*O*-methylamentoflavone, ginkgetin, tri-*O*-methylamentoflavone, and tetra-*O*-methylamentoflavone [314]; GB-2a can inhibit the formation of melanin [315]; studies have shown that isoginkgetin is an inhibitor of mRNA splicing [316]; and chamaejasmine and ginkgetin can improve chronic dermatitis through anti-inflammatory effects [317,318,319]. All of the above are the pharmacological effects discovered and studied in recent years for biflavonoids, indicating that biflavonoids have great developmental prospects.

## 6. Pharmacokinetics

LC-MS/MS is a sensitive method used in pharmacokinetics, and it is also used in the pharmacokinetics of biflavonoids. It is used in the study of amentoflavone pharmacokinetics by different drug intake modes, including oral gavage (p.o.), intravenous (i.v.), or intraperitoneal (i.p.) injection in rat models. As a result, 90.7% ± 8.3% of the total amount of amentoflavone (300 mg/kg) by p.o., 73.2% ± 6.29% of amentoflavone (10 mg/kg) by i.v., and 70.2% ± 5.18% of the total amentoflavone (10 mg/kg) by i.p. could be detected. The total amentoflavone was found to circulate as conjugated metabolites in the plasma of rats after different modes of administration [320].

Amentoflavone was used as the standard of the study of pharmacokinetics of biflavonoids in LC-MS/MS. For instance, the pharmacokinetics of total hinokiflavone in rat plasma was studied by LC-MS/MS. It was discovered that T_1/2_ was 6.10 ± 1.86 h [321].

However, there are other ways to calculate the main index of pharmacokinetics. The main components of *Platycladus orientalis* leaf extract include amentoflavone and hinokiflavone. Therefore, their pharmacokinetics in the plasma of a rat model were evaluated by UFLC-MS/MS. Their T_1/2_ and Tmax were 2.60 ± 1.34 h and 1.5 ± 0.00 h (amentoflavone), and 2.11 ± 0.29 h and 1.92 ± 0.20 h (hinokiflavone), respectively [322]. All the pharmacokinetics data of biflavonoids were showed in Table 12.

## 7. The Biosynthesis and Synthesis of Biflavonoids

### 7.1. The Biosynthesis of Biflavonoids

**Figure 13:**
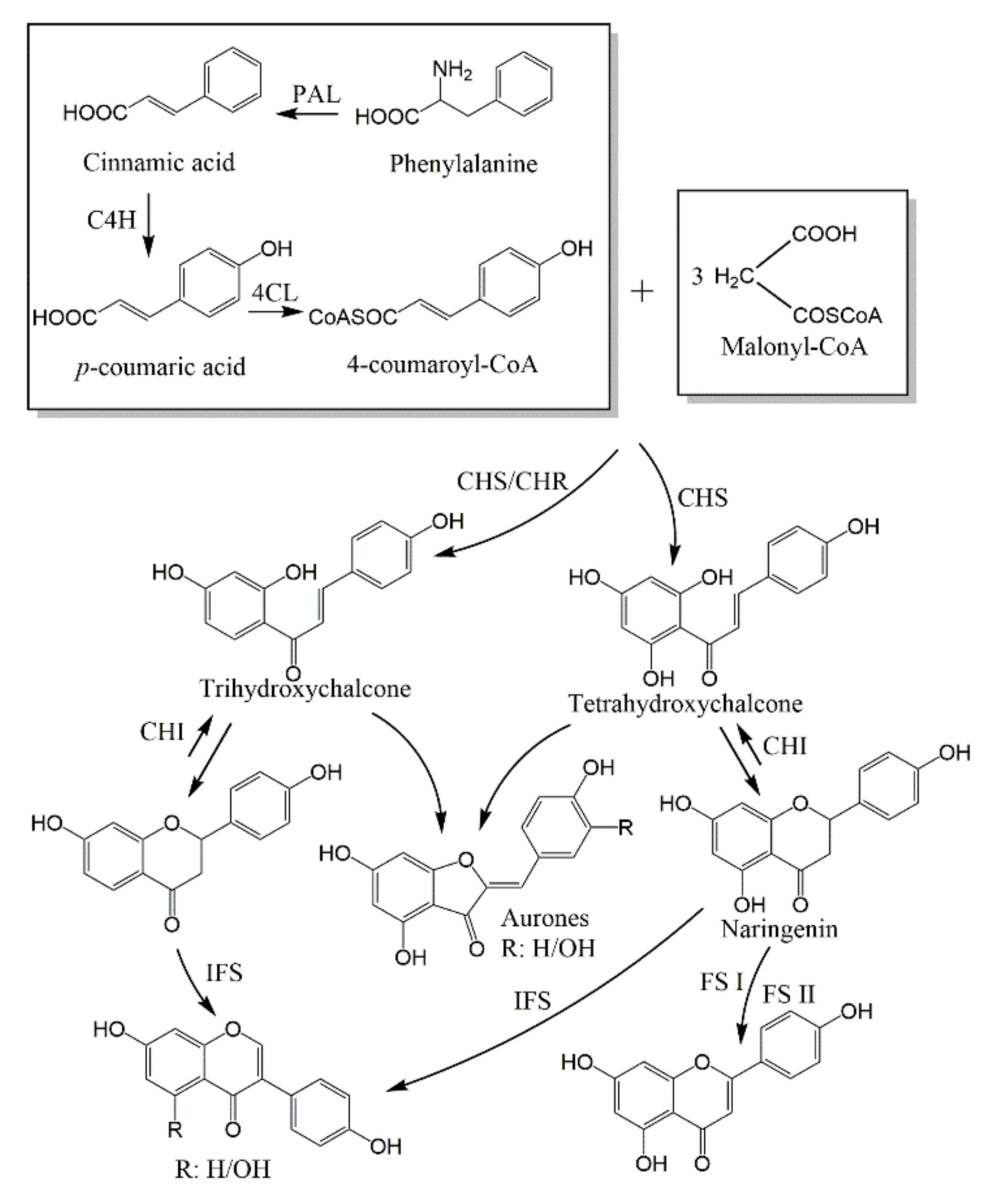
The biosynthesis of flavonoids. Enzyme names are abbreviated as follows: cinnamate-4-hydroxylase (C4H), chalcone isomerase (CHI), chalcone reductase (CHR), chalcone synthase(CHS), 4-coumaroyl:CoA-ligase (4CL), flavone synthase (FS I and FS II), isoflavone synthase (IFS), and Phe ammonia-lyase (PAL).

There were few references about the biosynthesis of biflavonoids, but it involves the oxidative coupling of two flavonoid units; therefore, the biosynthesis of flavonoids was a significant step to shape biflavonoids in plants. Alzand et al. [323] had reviewed the major pathways of flavonoid biosynthesis. Starting from phenylpropanoid metabolism and then giving the chalcone (trihydroxychalcone and tetrahydroxychalcone). The tetrahydroxychalcone is isomerised to naringenin, a key intermediate, which can transform to several end-flavonoids (Figure 13).

Furthermore, promoting the biosynthesis of biflavonoids can improve the yield of biflavonoids in plants by changing different catalytic enzymes or elicitors. Kicia Karinne Pereira Gromes-Copeland et al. [324] had converted the elicitors of 30 g/L of sucrose and 5 mg/L of 2,4-dichlorophenoxyacetic acid in *Poincianella pyramidalic*. Providing a higher accumulation of amentoflavone (16.44 mg/L) and agathisflavone (0.58 mg/L). Subsequently, they found that the amentoflavone biosynthesis is superior to agatisflavone. It seems to be related to the linkage type between two flavonoid units.

### 7.2. The Synthesis of Biflavonoids

Biflavonoids have great medicinal value and great development prospects. Therefore, the quantity needed in treatment and research will increase. However, it is impossible to obtain a large number of single and high-quality biflavonoids by simply extracting and separating the biflavonoids. In the process of synthesizing biflavonoids, Xue Ying et al. [325] reviewed the previous synthesis methods of biflavonoids in 2010, compared the differences between the various methods, and concluded that the synthesis method of biflavonoids is mainly to synthesize a flavonoid monomer. Then, two molecules of flavonoids coupled with boron-containing flavonoids are chosen by Suzuki or iodide-biflavonoids to obtain the final product, or the two molecules are coupled with the catalyst. The related C-C biosynthesis and reverse synthesis analysis, and the Ullmann ether condensation reaction of C-*O*-C, are also introduced. In the case of the literature that has been previously summarized, this review will conduct a general analysis of the new biflavonoid synthesis method, and compare the old method with the new one, so that readers can be more intuitive.

**Scheme 1:**
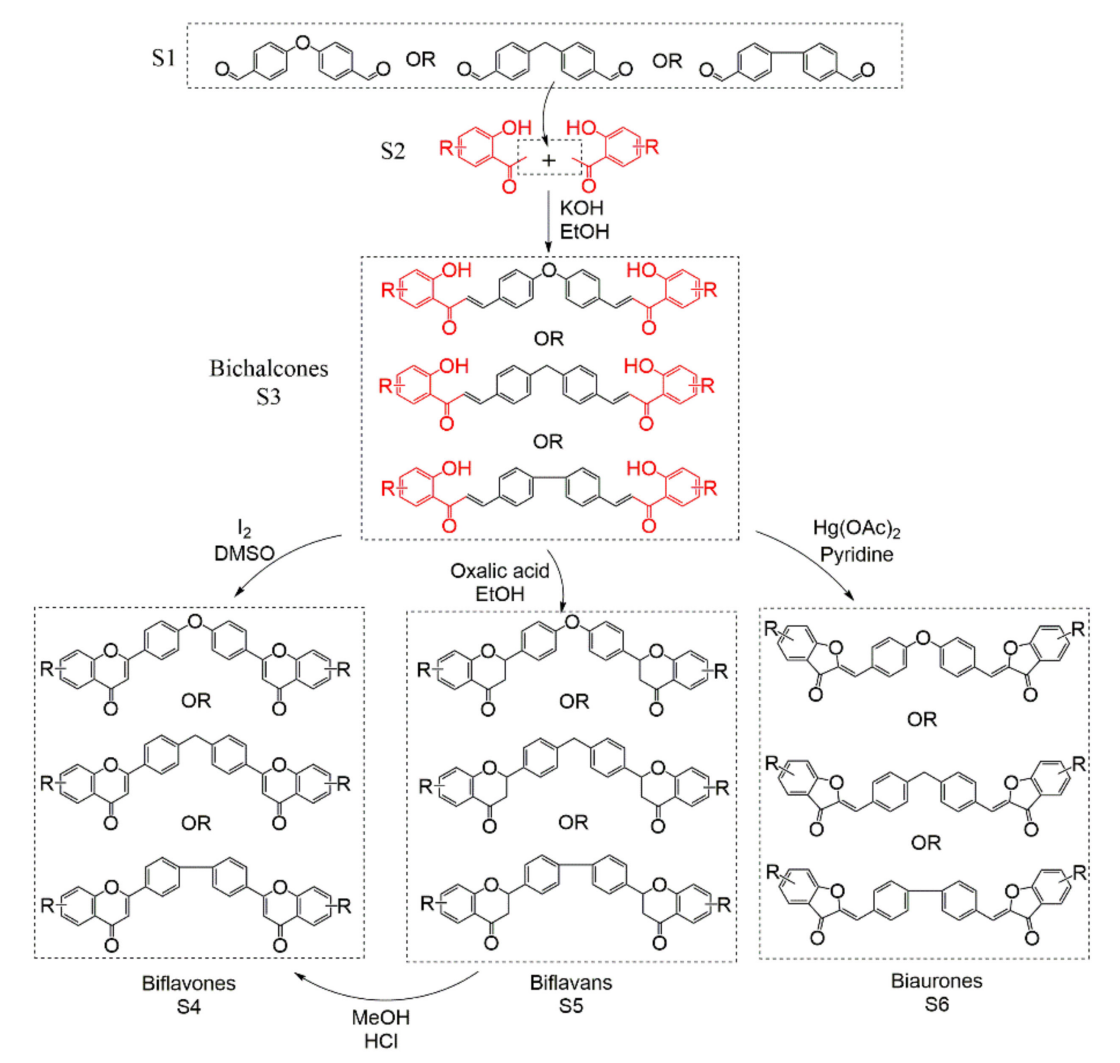
Total synthesis of C-*O*-C, C-C-C, and C-C biflavonoids.

Until 2017, the syntheses of biflavonoids were the construction of a biflavonoid skeleton, and different types of dimers were synthesized under different synthesis conditions. First, the biflavonoid skeleton, bichalcones (S3) is obtained by Claisen–Schmidt aldol condensation from the different dialdehyde molecules (S1) with the corresponding acetophenone (S2). Second, the bichalcone skeleton can obtain biflavones (S4), through iodine-mediated or produce biaurones (S6) by mercury acetate oxidation [27]. Biflavans can be obtained by oxalic acid with EtOH [326], but biflavans will change to biflavones in MeOH with HCl [327]. (Scheme 1). These methods can synthesize different types of biflavonoids as long as different dialdehydes can be provided. For example, the dialdehydes S1 are 4,4′-biphenyldicarboxaldehyde, 4,4′-diaryletherdicarboxaldehyde, or 4,4′-bitoluenedicarboxaldehyde, and the biflanonoids are C-C, C-*O*-C, or C-C-C. It can be said that this Claisen–Schmidt aldol condensation of dialdehyde and acetophenone can be the synthesis route of most symmetric biflavonoids. According to the difference in the final product, bichalcones, biflavones, biflavans, and biaurones can also be obtained by autonomously controlling the conditions.

**Figure 14:**
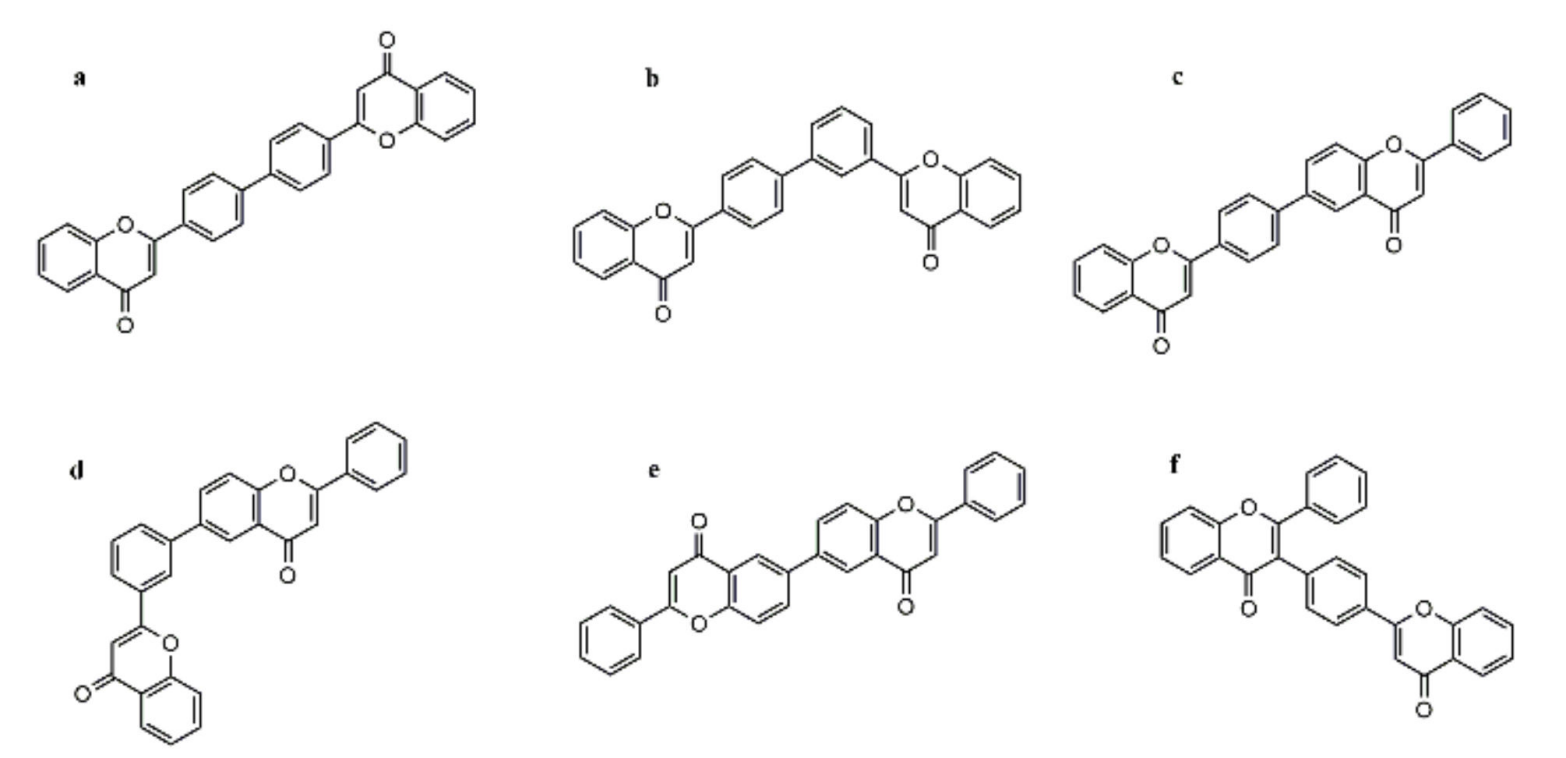
The structure of C-C 4′-4′ linkage biflavonoids.

Due to the large number and types of biflavonoids connected to C-C, there are many related synthetic studies. Among them, Chen et al., achieved the synthesis of C-C biflavonoids through the construction of two flavonoid analogs in 2006: one flavonoid analog substituted by a halogen atom (bromide), and the other substituted by a group coupled by a transition metal-catalyzed cross-coupling method, namely two typical methods: the Suzuki coupling reaction and the Stille coupling reaction. The two flavonoid monomers are connected through the biaryl group. In addition, they synthesized a series of C-C 4’-4’linkage biflavonoids a–f and compared the inhibition of sPLA2-IIA among them. Amentoflavone and ochnaflavone were used as controls. Subsequently, they found that the inhibitory potency of the synthesis biflavonoid a(IC50 = 3.0 + 0.9 M) was slightly better than ochnaflavone(IC50 = 3.5 + 0.6 M), the biflavonoids b(IC50 = 15.5 + 3.7 M), d(IC50 = 19.9 + 4.6 M), and f(IC50 = 23.2 + 3.1 M) possessed the comparative inhibitory potency with amentoflavone(IC50 = 23.8 + 3.4 M) [328]. The C-C 4’-4’ linkage biflavonoids a–f are shown in Figure 14.

**Scheme 2:**
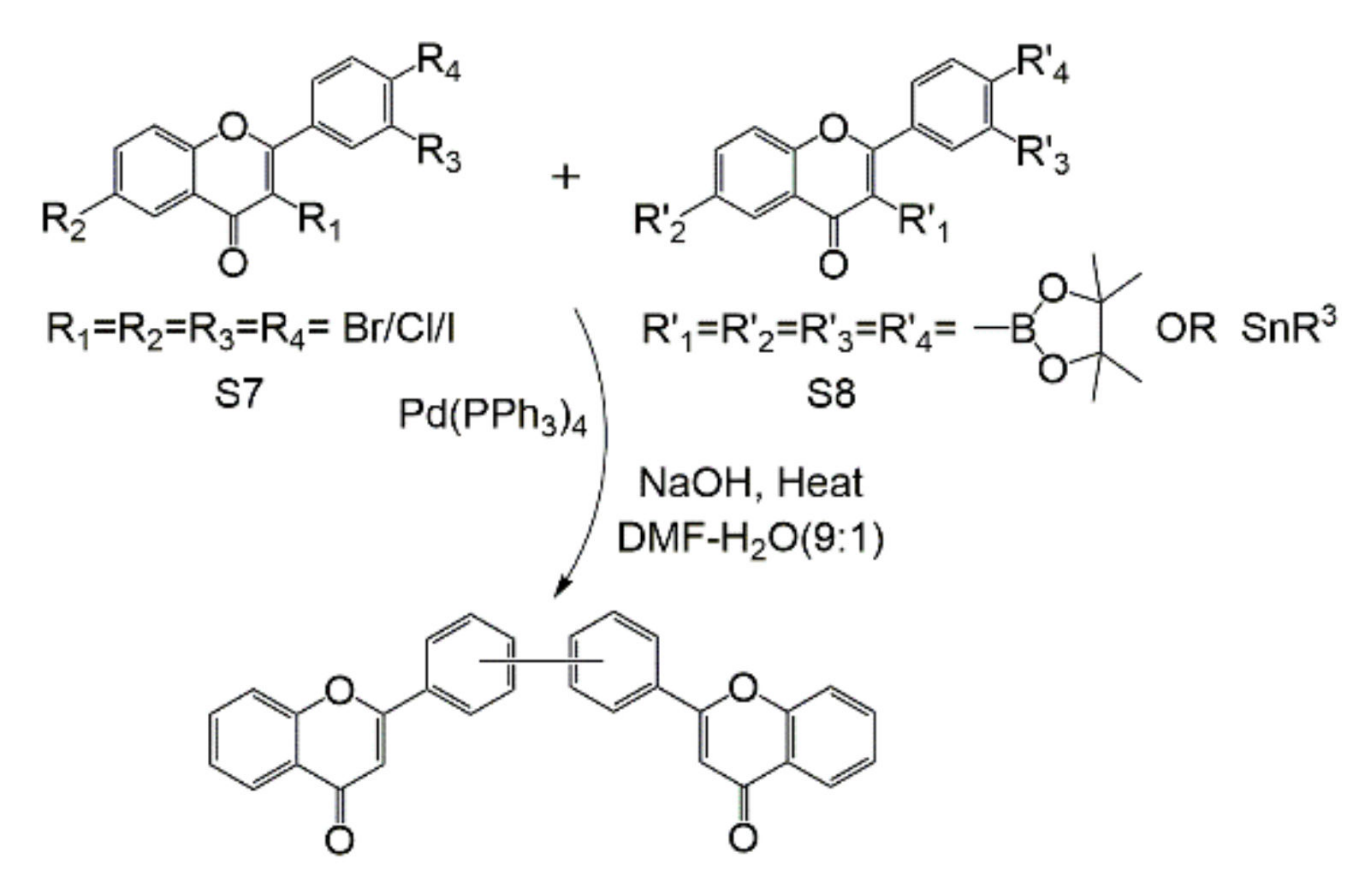
The synthesis of C-C biflavonoids.

However, due to the low yield of the above method, it is impossible to obtain high-yield biflavonoids on a large scale; as a result, researchers have found other ways to synthesize C-C type biflavonoids. Brominated, iominated, or chlorine substituted flavones (S7) and commercially available bis(pinacolato) diboron are reacted to obtain the corresponding pinacolato boronates (S8), and then S8 (120 mol%) and S7 under standard conditions (Pd(PPh_3_)_4_ (5 mol%), NaOH (400 mol%), and DMF-water (9:1), 100 °C) are reacted to obtain C-C biflavones. R1, R2, R3, R4, R′1, R′2, R′3, and R′4 are the positions attacked by brominated, iominated, chlorine, or bis(pinacolato) diboron. Moon et al., adjusted the reaction conditions to catalytic PdCl_2_(dppf) and K_2_CO_3_ in DMF at 90 °C, to reduce the loss of products [329] (Scheme 2).

According to the above method, Lim et al., processed chrysin into the precursor product required for the reaction and then performed the relevant synthesis under standard conditions to obtain a C-C (6-6′′) anti-inflammatory biflavonoid G168 [330], which had the potency of inhibiting COX-2 mediated PGE2 production. For G168, the IC_50_ value of inhibiting PGE2 production and againsting iNOS-mediated NO production were 0.1 μM and 50 μM. Furthermore, 5 mg/kg G168 was able to inhibit the paw edema in mice (30% inhibition) and 1~5 mg/kg G168 had the capacity to inhibit writhing in mice (57.3~82.9%). It has been proven that this method can synthesize amentoflavone-type biflavonoids [331] and Wikstrol A and B [332].

**Scheme 3:**
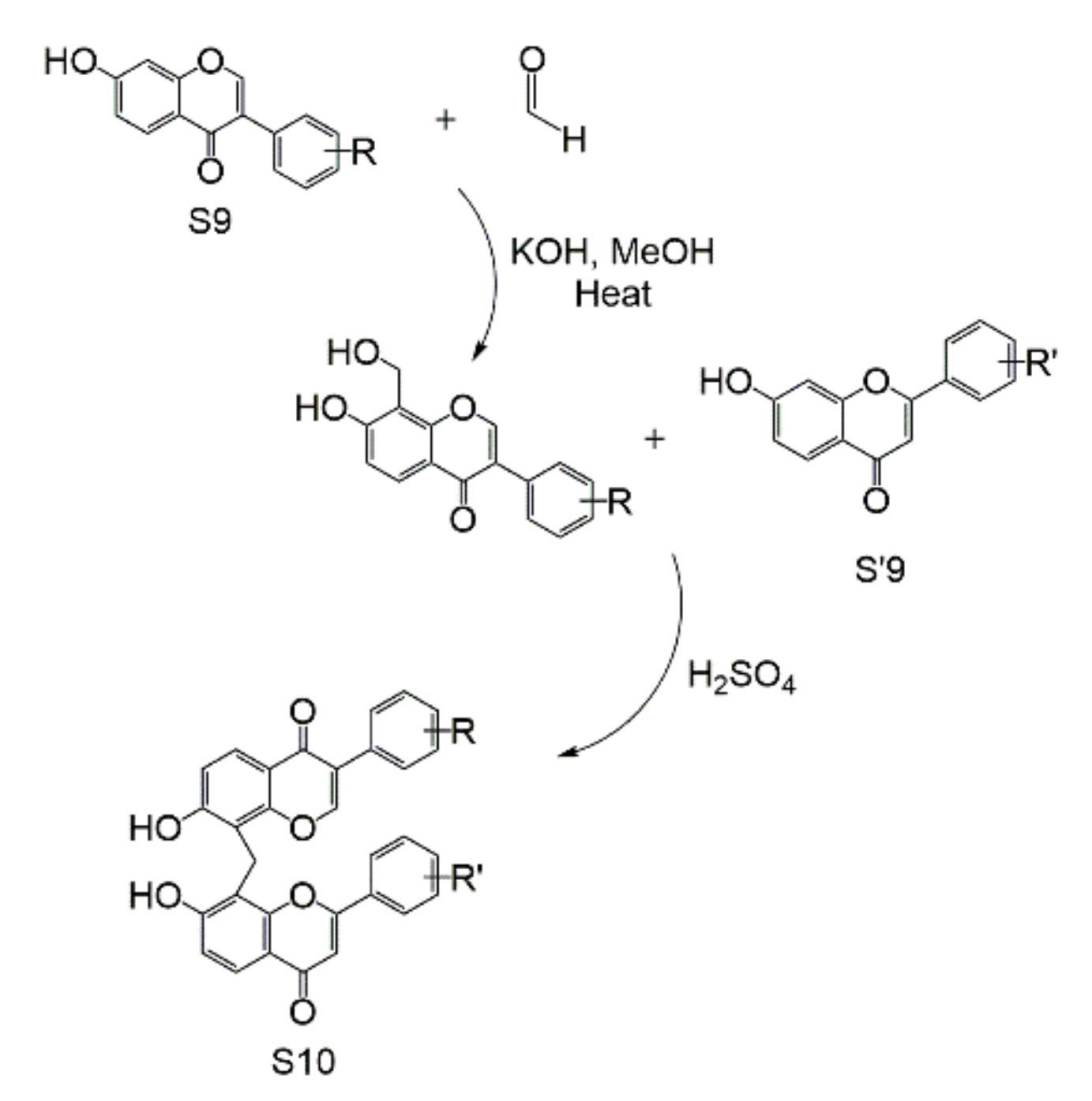
The synthesis of C-C-C biflavonoids.

In addition to the synthesis shown in Scheme 1, C-C-C-type biflavonoids can be obtained by the Ullmann condensation reaction of the corresponding flavonoid monomers; assuming the relevant conditions are controlled, and the yield is generally high. For instance, the flavonoid monomer chrysin was used to obtain 7-hydroxy-8-hydroxymethyl-4′-methoxyisoflavonoid [333]. Therefore, the synthesis of C-C-C can be summarized as follows: flavone monomer S9 reacts with formaldehyde to form an intermediate, and then it reacts with another flavone monomer S’9 to form C-C-C type biflavone S10 (Scheme 3). However, the yield of the synthesis in the above conditions is low. Thus, Xue Ying et al., modified the method in 2010; they used daidzein as the raw material, the catalyst was concentrated sulfuric acid, and the feed ratio of the raw material was daidzein. When the amount of catalyst was 10% of the molar ratio of daidzein, and the reaction temperature was 80 °C for 24 h, the highest yield of daidzein biflavonoid derivative was obtained [334]. Later, in 2011, Xue Ying et al., further improved the method: using 9% Lewis acid as a catalyst, isoflavones and formaldehyde as raw materials, and controlling the reaction temperature to 90 °C for 20 h; the yield can reach 82~85% [335].

**Scheme 4:**
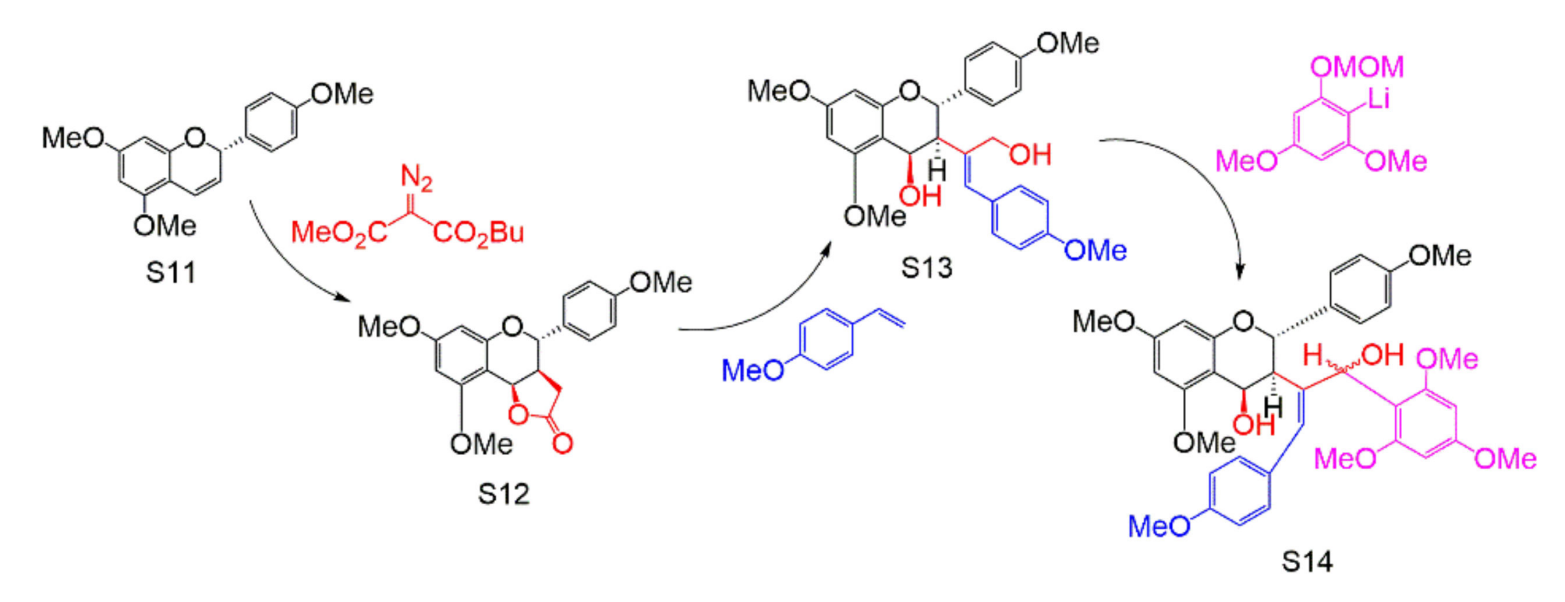
The de novo synthesis of biflavonoids.

In 2015, Baron and Mead first synthesized 3-benzylidene-dihydrofurochromen2-ones (S14), a flavan-chalcone type biflavonoid [336]. This was the first time de novo synthesis was attempted. The raw material of this synthetic route was flavonoids and chromene (S11). In the presence of catalytic Rh2(S-TBSP)4, the researchers treated S11 with a diazo derivative to obtain the donor-acceptor cyclopropane, and then used Sn(OTf)_2_ to rearrange the donor-acceptor cyclopropane to obtain the α-carbomethoxy lactone (S12). Then, removal from the hydroxyl protection and treatment with enolate lithium will result in a mixture of stereoisomers with a high hydroxyl alcohol ratio. The alcohol base was protected by TESCl and oxidized by DDQ to selectively oxidize the protected allyl alcohol to obtain aldehyde S13. After adding the aryl lithium reagent, 71% of the final product S14 was obtained, which was an inseparable isomer mixture, but it had all of the functions of target biflavonoids (Scheme 4). Compared with the synthetic methods of Scheme 1, the required conditions are more difficult to control, but it may become one kind of synthetic method that can control the separation of intermediate stereoisomers to better obtain a pure single product and heterobiflavonoids with different types of flavonoid monomers.

## 8. Conclusions

In recent years, the method of extracting active ingredients from herbs and using them in research experiments has been a key research direction, and also a huge challenge. As the components in plants are complex, there are many metabolites, and current extraction and separation technologies are still insufficient. A suitable method to efficiently extract, purify, and apply the required active ingredients is the goal we need to achieve. There are many kinds of biflavonoids, and there is an increasing number of synthetic biflavonoids; they are used as anti-inflammatory and antioxidant therapeutics, as treatments for Alzheimer’s disease and Parkinson’s disease, and for other therapeutic applications. Their use is more significant in anticancer and antiviral treatment. Moreover, Qiu-xia et al. [337] developed and applied amentoflavone based on antisolvent freeze-drying technology, and studied its stability during storage and the stable type of drug efficacy to solve the problem of poor water solubility of amentoflavone micropowder, and improve the oral availability of the drug. In particular, *Ginkgo biloba* has been proven to be useful in clinical treatment [338]. In summary, there is still much room for developing the pharmacology and synthesizing of biflavonoids, but there is a large gap in the research on dosage forms that needs to be supplemented by additional research. This review mainly provides a more detailed report on the classification, pharmacology, pharmacokinetics, synthesis, and other aspects of biflavonoids, to assist researchers in exploring biflavonoids.

## Figures and Tables

**Table 1:** The 2-3′′, 2′-2′′′, 2′-6′′, 2′-8′′, and 3-3′′ subtypes of biflavonoids.

Subtype	No.	Compounds Name	Monomer Type	Origin (Family *)	References
2-3′′	**1**	Linobiflavonoid	AH	Thym	[1]
2′-2′′′	**2**	I-3,II-3,I-5,II-5,I-7,II-7,I-4′,II-4′-octahydroxy[I-2′,II-2′]biflavone	BB	Clus	[3]
	**3**	I-3,II-3,I-5,II-5,I-7,II-7,I-4′,II-4′-octamethoxy[I-2′,II-2′]biflavone	BB	Clus	[3]
	**4**	3,3′′-di-*O*-α-L-rhamnopyranoside,2′,2′′′-bimyricetin	BB	Myrt	[4]
	**5**	Acuminatanol	AA	Anac	[5]
	**6**	Theasinensin A	AA	Thea	[6]
	**7**	Theasinensin B	AA	Thea	[6]
2′-6′′	**8**	2′,6′′-Biapignin	BB	Sela	[7]
2′-8′′	**9**	2′,8′′-Biapignin	BB	Sela	[7]
3-3′′	**10**	Chamaejasmine	AA	Thym, Legu	[8]
	**11**	7-methoxychamaejasmin	AA	Thym	[8]
	**12**	Ruixianglangdu B	AA	Thym	[8]
	**13**	Isosikokianin	AA	Thym	[8]
	**14**	7,4′,7′′,4′′-tetramethylisochamaejasmin	AA	Ochn	[9]
	**15**	7,7′′-di-*O*-methylchamaejasmin	AA	Legu	[10]
	**16**	Campylospermone A	AA	Ochn	[10,11]
	**17**	Campylospermone B	AA	Ochn	[11]
	**18**	Isochamaejasmine	AA	Thym	[12]
	**19**	7,7′′-di-*O*-glucosylisochamaejasmin	AA	Thym	[13]
	**20**	Neochamaejasmin A	AA	Thym	[8]
	**21**	Chamaejasmenin B	AA	Thym	[8]
	**22**	Chamaejasmenin C	AA	Thym	[8]
	**23**	Sikokianin A	AA	Thym	[8]
	**24**	7-methoxyneochamaejasmin A	AA	Thym	[8]
	**25**	Chamaejasmenin D	AA	Thym	[8]
	**26**	Isoneochamaejasmin A	AA	Thym	[8]
	**27**	Isochamaejasmine B	AA	Thym	[8]
	**28**	Neochamaejasmin B	AA	Thym	[8]
	**29**	Sikokianin B	AA	Thym	[12]
	**30**	Chamaejasmenin A	AA	Thym	[8]
	**31**	Sikokianin C	AA	Thym	[8,12]
	**32**	Ruixianglangdu A	AA	Thym	[8]
	**33**	Isosikokianin A	AA	Thym	[8]
	**34**	Asteryomenin	AA	Aste	[14]
	**35**	Wikstaiwanone C	AA	Thym	[12]
	**36**	Sikokianin D	AA	Thym	[15]
	**37**	2′′′-dehydroxy-2,2′′-bisteppogenin	AA	Thym	[1]
	**38**	2,2′′-bisteppogenin 7-*O*-β-glucopyranoside	AA	Thym	[1]
	**39**	2,2′′-bisteppogenin	AA	Thym	[1]
	**40**	Apigenil-(I-3,II-3)-naringenin	AA	Legu	[1]
	**41**	6-aminoacryloylchamaejasmin	AA	Legu, Thym	[1,16]
	**42**	Ormocarpin	AA	Legu	[1,13,17]
	**43**	(−)-7,7′′-di-*O*-glucosylchamaejasmin	AA	Legu	[1,13]
	**44**	(−)-(2S, 3S, 2′′S, 3′′R)-7-*O*-glucosylchamaejasmin	AA	Legu	[1]
	**45**	(2S, 3R, 2′′S, 3′′R)-7-*O*-glucosylchamaejasmin	AA	Legu	[1,17]
	**46**	Campilospermone B	AA	Ochn	[1]
	**47**	Neochamaejasmin C	AA	Thym	[1]
	**48**	7-methoxyneochamaejasmin B	AA	Thym	[1]
	**49**	2′′′-dehydroxy-2,2′′-bisteppogenin 7-*O*-β-glucopyranoside	AA	Thym	[1]
	**50**	3′′-epidiphysin	FF	Legu	[1]
	**51**	5,5′′-di-*O*-methyldiphysin	FF	Legu	[1,17]
	**52**	Diphysin	FF	Legu	[1]
	**53**	2,3-didehydro-(+)-chamaejasmin	AA	Thym	[16]
	**54**	3,3′-biliquiritigenin	AA	Ochn, Legu	[17]
	**55**	Euchamaejasmin A	AA	Thym	[18]
	**56**	7-*O*-β-D-glucopyranoside-diphysin	FF	Legu	[17]
	**57**	3-Epimer, 5,5′-dideoxy-diphysin	FF	Anac	[19]
	**58**	3,3′-diepimer, 5,5′-dideoxy-diphysin	FF	Anac	[19]
	**59**	6′′′-hydroxylophirone	AG	Ochn	[20]

* Anac: Anacardiaceae; Aste: Asteraceae; Clus: Clusiaceae; Legu: Leguminosae; Myrt: Myrtaceae; Ochn: Ochnaceae; Sela: Selaginellaceae; Thea: Theaceae; and Thym: Thymelaeaceae.

**Table 2:** The 3-3′′′, 3′-3′′′, 3′-4′′′, 3′-5′′, 3-6′′, 3′-6′′, 3-7′′, and 3′-7′′ subtypes of biflavonoids.

Subtype	No.	Compounds Name	Monomer Type	Origin (Family *)	References
3-3′′′	**60**	Taiwaniaflavone	BB	Cupr, Sela, Taxo	[21,22,23]
	**61**	7-*O*-methyltaiwaniaflavone	BB	Taxo	[23]
	**62**	4′′′,7-di-*O*-methyltaiwaniaflavone	BB	Taxo	[23]
	**63**	Lupinabisone A	AB	Legu	[24]
3′-3′′′	**64**	2,3-dihydro-3′,3′′′-biapigenin	BB	Sela	[7]
	**65**	3′,3′′-binaringenin	BB	Sela	[7,25]
	**66**	Thuidinin	BB	Thui	[25]
	**67**	Kudzuisoflavone A	EE	Faba	[26]
3′-4′′′	**68**	Chrysocauloflavone III	AB	Sela	[7]
	**69**	Japoflavone D	BB	Capr	[27]
3′-5′′	**70**	Aulacomniumbiaureusidin	HH	Aula	[28]
3-6′′	**71**	Daphnodorin K1	AB	Thym	[29]
	**72**	Daphnodorin K2	AB	Thym	[29]
	**73**	Wikstaiwanone A	AB	Thym	[12]
	**74**	Wikstaiwanone B	AB	Thym	[12]
	**75**	Stephaflavone A	BB	Meni	[30]
	**76**	Stephaflavone B	BB	Meni	[30]
	**77**	Isomanniflavone	AA	Clus	[1]
	**78**	Ridiculuflavone	BB	Aris	[1]
	**79**	Afzelone B	AA	Ochn	[31]
	**80**	Ridiculuflavone A	BB	Aris	[32,33]
	**81**	Ridiculuflavone B	BB	Aris	[32,33]
	**82**	Ridiculuflavone D	BB	Aris	[32]
	**83**	Ridiculuflavone C	BB	Aris	[32]
	**84**	4′′′,5,5′′,7′′-tetrahydroxy-3′′′,4′,7-trimethoxy-3,6′′-biflavone	BB	Aris	[32,34]
3′-6′′	**85**	Robustaflavone	BB	Anac, Arau, Clus, Sela	[35,36]
	**86**	7′′-*O*-methylrobustaflavone	BB	Sela	[7]
	**87**	4′-*O*-methylrobustaflavone	BB	Sela	[7,35,37]
	**88**	7,4′-di-*O*-methylrobustaflavone	BB	Sela	[7,38]
	**89**	4′,4′′′-di-*O*-methylrobustaflvone	BB	Sela	[7,39]
	**90**	4′,7′′-di-*O*-methylrobustaflavone	BB	Sela	[7,37]
	**91**	7,4′,4′′′-tri-*O*-methylrobustaflvone	BB	Sela	[7,39]
	**92**	Imbricataflavone A	AB	Podo, Sela	[7,35,40]
	**93**	Caesalflavone	AB	Legu, Sela	[7,41,42]
	**94**	Uncinatabiflavone D	AB	Sela	[7,43,44]
	**95**	7,4′,7′′-tri-*O*-methyl-2,3-dihydrorobustaflavone	AB	Sela	[7,39]
	**96**	5-*O*-methyl-2,3-dihydrorobustaflavone	AB	Sela	[7,42]
	**97**	Macrophylloflavone	AB	Sela	[45]
	**98**	2,3-dihydrorobustaflavone 7,7′′-dimethyl ether	AB	Sela, Thym	[1]
	**99**	Imbricataflavone B	AB	Podo	[40]
	**100**	4′-*O*-methyl-2′′,3′′-dihydrorobustaflavone	AB	Sela, Thym	[7,43]
	**101**	7,4′-di-*O*-methyl-2′′,3′′-dihydrorobustaflvone	AB	Arau, Sela, Thym	[7,37]
	**102**	4′,7′′-di-*O*-methyl-2′′,3′′-dihydrorobustaflavone	AB	Arau, Sela, Thym	[36]
	**103**	7,4′,7′′-tri-*O*-methyl-2′′,3′′-dihydrorobustaflavone	AB	Sela, Thym	[7,37]
	**104**	Robustaflavanone	AA	Sela, Thym	[7,38]
	**105**	Uncinatabiflavone A	AA	Sela	[7,43,44]
	**106**	Uncinatabiflavone B	AA	Sela	[7,43,44]
	**107**	Uncinatabiflavone C	AA	Sela	[7,43,44]
	**108**	7,4′,7′′-tri-*O*-methyl-2,3,2′′,3′′-tetrahydrorobustaflavone	AA	Sela	[7,35]
	**109**	Abiesin	BB	Pina	[46]
	**110**	5′-hydroxyrobustaflavone	BB	Hylo	[47]
	**111**	2′′,3′′-dihydro-5′′′-hydroxyrobustaflavone	AB	Mnia	[48]
	**112**	5′,6′′-biluteolin	BB	Hylo, Dicr	[49]
	**113**	2,3-dihydro-5′,6′′-biluteolin	AB	Dicr	[49]
	**114**	2′′,3′′-dihydro-5′,6′′-biluteolin	AB	Mnia	[50]
3-7′′	**115**	5,5′′,6′′,7,8-pentahydroxy-2,2′′-bis(p-hydroxyphenyl)-4H,4′′H(3,7′′-bichromene)-4,4′′-dione	BB	Anac	[51]
3′-7′′	**116**	Lophirone M	AB	Ochn	[52]
	**117**	Lophirone M hexa-acetate	AB	Ochn	[52]

* Aula: Aulacomniaceae; Arau: Araucariaceae; Aris: Aristolochiaceae; Capr: Caprifoliaceae; Cupr: Cupressaceae; Faba: Fabaceae; Meni: Menispermaceae; Podo: Podocarpaceae; Taxa: Taxaceae; Taxo: Taxodiaceae; and Thui: Thuidiaceae.

**Table 3:** The 3-8′′ subtype of biflavonoids.

No.	Compounds Name	Monomer Type	Origin (Family *)	References
**118**	Garciniaflavone E	AB	Clus	[1]
**119**	Garciniaflavone F	AB	Clus	[1]
**120**	Morelloflavone-7′′-*O*-β-D-glucosíde	BB	Clus	[1]
**121**	(+)-4′′′-*O*-methylmorelloflavone	AB	Clus	[1]
**122**	Biapigenin	BB	Clus	[1]
**123**	4′′′-*O*-methyl-I3,II8-binaringenin	AA	Clus	[1,53]
**124**	Volkensiflavone	AB	Clus	[54]
**125**	Morelloflavone	AB	Clus	[55]
**126**	Spicataside	AB	Clus	[55]
**127**	Fukugiside	AB	Clus	[55]
**128**	3′′′-*O*-methylfukugetin	AB	Clus	[1]
**129**	Garcinianin	AB	Clus	[55,56,57]
**130**	Madrunoudeaside	AB	Clus	[58]
**131**	Morelloflavone-7′-sulfate	AB	Clus	[59]
**132**	(2R,3S)-morelloflavone	AB	Mora	[60]
**133**	7,4′,7′′,3′′′,4′′′-penta-*O*-acetylmorelloflavone	BB	Mora	[60]
**134**	7,4′,7′′,3′′′,4′′′-penta-*O*-methylmorelloflavone	BB	Mora	[60]
**135**	7,4′,7′′,3′′′,4′′′-penta-*O*-butanoylmorelloflavone	BB	Mora	[60]
**136**	Talbotaflavone	BB	Clus	[1,61]
**137**	Balsamiside A	AB	Bals	[62]
**138**	Balsamiside B	AB	Bals	[62]
**139**	Balsamiside C	AB	Bals	[62]
**140**	Balsamiside D	AB	Bals	[62]
**141**	Daphnodorin D1	AB	Thym	[29]
**142**	Daphnodorin D2	AB	Thym	[29]
**143**	Wikstrol A	AB	Thym	[63]
**144**	Wikstrol B	AB	Thym	[63]
**145**	II-3,I-5, II-5,II-7,I-4′,II-4′-hexahydroxy-(I-3,II-8)-flavonylflavanonol	BB	Clus	[1]
**146**	GB-1a	AA	Clus	[54,64]
**147**	GB-2a	AB	Clus	[65]
**148**	GB-1a-7′′-*O*-glycoside	AA	Clus	[55]
**149**	Xanthochymuside	AA	Clus	[55,66]
**150**	Kolaflavanone	AA	Clus	[55,64]
**151**	GB-1	AA	Clus	[55,64]
**152**	GB-2	AA	Clus	[55,64]
**153**	Manniflavanone	AA	Clus	[54,64]
**154**	GB-2a-II-4′-OMe	BB	Clus	[65]
**155**	Buchananiflavone	AA	Clus	[1,67]
**156**	Manniflavone-7′′-*O*-β-D-glucopyranoside	AB	Clus	[1]
**157**	(2R,3S,2′′R)3,8′′-binaringenin-7′′-*O*-β-glucoside	AA	Clus	[1,68]
**158**	(2R, 3S, 2′′R, 3′′R)GB1-7′′-*O*-β-glucoside	AA	Clus	[1,68]
**159**	Ent-naringenil-(I-3α,II-8)-4′-*O*-metilnaringenin	BB	Clus	[1,53]
**160**	3,8′′-biapigenin	BB	Poly, Clus	[69,70]
**161**	Sumaflavone	BB	Anac	[7,71]
**162**	4′-methoxydaphnodorin D1	AB	Thym	[72]
**163**	4′-methoxydaphnodorin D2	AB	Thym	[72]
**164**	Pancibiflavonol	AB	Clus	[73]
**165**	Volkensiflavone 7-sulfate	AB	Clus	[74]
**166**	8-(3′,4′,5,7-tetrahydroxyflavanon-3-yl)-4′,5,7-trihydroxyflavone	AB	Clus	[75]
**167**	GB-3	AA	Clus	[64]
**168**	GB-4	AA	Clus	[76]
**169**	GB-2b	AA	Clus	[64]
**170**	GB-4a	AA	Clus	[76]
**171**	4′′′-*O*-methylfukugetin	AB	Clus	[77]
**172**	Lupinalbisone B	AB	Legu	[24]

* Bals: Balsaminaceae; Dicr: Dicranaceae; Hylo: Hylocomiaceae Mnia: Mniaceae; Mora: Moraceae; Poly: Polygonaceae; and Pina: Pinaceae.

**Table 4:** The 3′-8′′ subtype of biflavonoids.

No.	Compounds Name	Monomer Type	Origin (Family *)	References
**173**	Amentoflavone	BB	Anac, Capr, Clus, Cupr, Gink, Pina, Podo, Pter, Sela, Taxa, Taxo	[2]
**174**	Isoginkgetin	BB	Gink, Sela	[2,7]
**175**	3′,8′′-biisokaempferide	BB	Pter, Vell	[1,2]
**176**	7,7′′-di-*O*-methylamentoflavone	BB	Arau, Cupr, Podo, Sela, Taxo	[2,7]
**177**	4′,7′′-di-*O*-methylamentoflavone	BB	Arau, Sela, Taxo	[2,7]
**178**	5′-methoxybilobetin	BB	Gink, Mnia	[2]
**179**	7,4,7′′,4′′′-tetra-*O*-methylamentoflavone	BB	Sela	[2,7]
**180**	7,4′,7′′-tri-*O*-methylamentoflavone	BB	Arau, Ceph, Cupr, Taxa	[2]
**181**	Acetyl ginkgetin	BB	Gink	[78]
**182**	Acetyl isogenkgetin	BB	Gink	[78]
**183**	Acetyl sciadopitysin	BB	Gink	[78]
**184**	6-C-methyl-7-*O*-methylamentoflavone	BB	Ceph	[79]
**185**	3′′′-hydroxy-4′′′,7-dimethylamentoflavone	BB	Aris, Taxa	[80]
**186**	Anacarduflavone	BB	Anac	[81]
**187**	Bilcarobetin	BB	Gink, Sela	[2,7]
**188**	CGY-1	BB	Lili	[82]
**189**	Ginkgetin	BB	Arau, Gink, Sela, Taxa, Taxo	[2,7]
**190**	Ginkgetin 7-*O*-D-glucopyranoside	BB	Gink	[83]
**191**	Heveaflavone	BB	Euph, Sela	[2,7]
**192**	7-*O*-gluamentoflavone	BB	Cupr, Gink	[84]
**193**	Isoginkgetin 7-*O*-D-glucopyranoside	BB	Gink	[83]
**194**	Amentoflavone 7′′-*O*-β-D-glucopyranoside	BB	Cupr, Gink	[84]
**195**	Kayaflavone	BB	Podo, Sela, Taxa, Taxo	[2,7]
**196**	Oliveriflavone	BB	Taxa	[79]
**197**	Oliveriflavone B	BB	Taxa	[85]
**198**	Oliveriflavone C	BB	Taxa	[85]
**199**	Podocarduflavone B	BB	Podo	[2]
**200**	Podocarpusflavone A	BB	Podo, Sela	[2,7]
**201**	Sciadopitysin	BB	Cupr, Gink, Podo, Taxa, Taxo	[2]
**202**	Sequoiaflavone	BB	Sela, Taxo	[2,7]
**203**	Sotetsuflavone	BB	Cyca, Sela, Taxo	[2,7]
**204**	Taiwaniaflavone A	BB	Ceph	[2]
**205**	Dulcisbiflavonoid A	BB	Clus	[59]
**206**	Putraflavone	BB	Euph, Podo	[1]
**207**	(2S,2′′S)-2,3-di-hydroisoginkgetin	AB	Cyca	[86]
**208**	2,3-dihydro-6-methylginkgetin	AB	Ceph	[1]
**209**	2,3-dihydrosciadopitysin	AB	Gink, Taxo	[83]
**210**	4′,7′′-di-*O*-methyl-2,3-dihydroamentoflavone	AB	Sela	[7]
**211**	(2S)-2,3-dihydroamentoflavone	AB	Sela	[7]
**212**	7,4′-di-*O*-methyl-2,3-dihydroamentoflavone	AB	Cupr, Sela	[7]
**213**	7,4′,7′′-tri-*O*-methyl-2,3-dihydroamentoflavone	AB	Arau, Sela, Taxa	[7]
**214**	2,3-dihydro-4-*O*-methylamentoflavone	AB	Cyca, Sela	[87]
**215**	(2S)-4′-*O*-methyl-2,3-tetrahydroamentoflavone	AB	Cyca, Sela	[7]
**216**	Garciniaflavone A	AB	Clus	[1,2]
**217**	Garciniaflavone B	AB	Clus	[1,2]
**218**	Garciniaflavone C	AB	Clus	[1,2]
**219**	Garciniaflavone D	AB	Clus	[1,2]
**220**	6′′-hydroxy-2,3-dihydroamentoflavone	AB	Sela	[7]
**221**	Selamariscina A	AB	Sela	[36]
**222**	2′′,3′′-dihydroamentoflavone	AB	Anac, Cyca, Sela, Taxo	[7]
**223**	4′-*O*-methyl-2′′,3′′-dihydroamentoflavone	AB	Cyca, Sela	[1,7]
**224**	(2S,2′′S)-2,3,2′′,3′′-tetrahydroisoginkgetin	AA	Arau, Cyca, Podo	[86]
**225**	(2S,2′′S)-2,3,2′′,3′′-tetrahydroamentoflavone	AA	Anac, Cyca, Sela	[7,86]
**226**	(2S,2′′S)-4′-*O*-methyl-2,3,2′′,3′′-tetrahydroamentoflavone	AA	Sela	[7]
**227**	Taxusbiflavone A	BB	Capr	[88]
**228**	3′′′-methoxyamentoflavone	BB	Anac	[89]
**229**	3′′′,5′-dihydroxyamentoflavone	BB	Dicr	[49]
**230**	(2S,2′′S)-3′,4′,4′′′,5,5′′,7′′-hexahydroxy-8,3′′′-biflavanone	AA	Anac	[81]
**231**	3′,5,5′′-trihydroxy-4′,4′′′,7′′-trimethoxy-8,3′′′-biflavanone	AA	Anac	[90]
**232**	5,5′′-dihydroxy-3′,4′,4′′′,7′′-tetramethoxy-8,3′′′-biflavanone	AA	Anac	[91]
**233**	Anacarduflavanone	AA	Anac	[92]

* Capr: Caprifoliaceae; Ceph: Cephalotaxaceae; Cyca: Cycadaceae; Euph: Euphorbiaceae; Gink: Ginkgoaceae; Lili: Liliaceae; Pter: Pteridiaceae; and Vell: Velloziaceae.

**Table 5:** The 4-6′′ subtype of biflavonoids.

No.	Compounds Name	Monomer Type	Origin (Family *)	References
**234**	Sarcandrone D	AA	Chlo	[1]
**235**	Procyanidin B5	AA	Dava, Malv, Pina, Rosa, Sapi	[93,94]
**236**	Epicatechin 3-*O*-gallate-(4β→6)-epicatechin 3-*O*-(4-hydroxybenzoate)	AA	Myro	[95]
**237**	3′-*O*-galloylprocyanidin B5	AA	Vita	[95,96]
**238**	3,3′-di-*O*-galloylprocyanidin B5	AA	Poly	[97]
**239**	Epigallocatechin 3-*O*-gallate-(4β→6)-epicatechin 3-*O*-gallate	AA	Thea	[98]
**240**	Epicatechin 3-*O*-gallate-(4β→6)-epigallocatechin 3-*O*-gallate	AA	Thea	[98]
**241**	Epigallocatechin-(4β→6)-epigallocatechin 3-*O*-gallate	AA	Cist	[99]
**242**	3,3′-di-*O*-galloylprodelphinidin B5	AA	Myri	[100]
**243**	Epiafzelechin 3-*O*-gallate-(4β→6)-epigallocatechin 3-*O*-gallate	AA	Thea	[98]
**244**	Procyanidin B6	AA	Cupr, Eric, Rosa	[93]
**245**	Procyanidin B7	AA	Cupr, Eric	[93]
**246**	Procyanidin B8	AA	Eric, Rosa, Sali	[93]
**247**	Ent-epicatechin-(4α→6)-ent-epicatechin	AA	Malp	[101]
**248**	Fisetinidol-(4β→6)-fisetinidol-4β-ol	AA	Legu	[102]
**249**	Fisetinidol-(4β→6)-fisetinidol	AA	Legu	[103]
**250**	Fisetinidol-(4β→6)-fisetinidol-4α-ol	AA	Legu	[102]
**251**	Fisetinidol-(4β→6)-ent-epifisetinidol	AA	Legu	[103]
**252**	Fisetinidol-(4α→6)-fisetinidol-4β-ol	AA	Legu	[102]
**253**	Fisetinidol-(4α→6)-fisetinidol	AA	Legu	[103]
**254**	Fisetinidol-(4α→6)-fisetinidol-4α-ol	AA	Legu	[102]
**255**	Fisetinidol-(4α→6)-ent-epifisetinidol	AA	Legu	[103]
**256**	Globiflorin 3B1	AA	Legu	[104]
**257**	Globiflorin 3B2	AA	Legu	[104]
**258**	Guibourtinidol-(4α→6)-afzelechin	AA	Legu	[105]
**259**	ent-Guibourtinidol-(4β→6)-catechin	AA	Rosa	[106]
**260**	Epicatechin-(4β→6)-epicatechin-(4β→2)-phloroglucinol	AA	Legu, Pina, Rosa	[107,108,109]
**261**	Guibourtinidol-(4α→6)-epicatechin-8-carboxylic acid	AA	Legu	[104]
**262**	Guibourtinidol-(4α→6)-catechin-8-carboxylic acid	AA	Legu	[104]
**263**	Epioritin-(4β→6)-epioritin-4α-ol	AA	Legu	[110]
**264**	Epioritin-(4β→6)-epioritin-4β-ol	AA	Legu	[110]
**265**	Epioritin-(4β→6)-oritin-4α-ol	AA	Legu	[111]
**266**	Epioritin-(4β→6)-ent-oritin-4α-ol	AA	Legu	[110]
**267**	Ent-Oritin-(4β→6)-epioritin-4α-ol	AA	Legu	[110]
**268**	Ent-Oritin-(4α→6)-epioritin-4α-ol	AA	Legu	[111]
**269**	Ent-Oritin-(4α→6)-epioritin-4β-ol	AA	Legu	[111]
**270**	Ent-Oritin-(4β→6)-oritin-4α-ol	AA	Legu	[110]
**271**	Ent-Oritin-(4α→6)-oritin-4α-ol	AA	Legu	[111]

* Chlo: Chloranthaceae; Cist: Cistaceae; Dava: Davalliaceae; Eric: Ericaceae; Malv: Malvaceae; Malp: Malpighiaceae; Myri: Myricaceae; Myro: Myrothamnaceae; Rosa: Rosaceae; Sali: Salicaceae; and Sapi: Sapindaceae.

**Table 6:** The 4-8′′ subtype of biflavonoids.

No.	Compounds Name	Monomer Type	Origin (Family *)	References
**272**	Juglbiflavone A	AB	Jugl	[112]
**273**	Sarcandrone	AA	Chlo	[1]
**274**	Procyanidin B2	AA	Aizo, Rosa, Sapi	[94,113,114]
**275**	Procyanidin B2 7′-xyloside	AA	Legu	[115]
**276**	3′-galloylprocyanidin B2	AA	Poly,Vita	[6]
**277**	3,3′-digalloylprocyanidin B2	AA	Poly, Thea, Rosa	[6,114]
**278**	3′-*O*-(3,4-di-*O*-methylgalloyl)procyanidin B2	AA	Poly	[94]
**279**	Epicatechin-(4α→8)-epicatechin	AA	Rosa	[116]
**280**	Procyanidin B1	AA	Legu	[114]
**281**	3′-(4-hydroxybenzoyl)procyanidin B1	AA	Hama	[117]
**282**	3-galloylprocyanidin B1	AA	Poly	[114,118]
**283**	3′-*O*-(1-hydroxy-6-oxo-2-cyclohexene-1-carboxylate)procyanidin B1	AA	Sali	[119]
**284**	Procyanidin B4	AA	Rosa	[113]
**285**	3-*O*-β-D-glucopyranoside, 3′-*O*-(6-*O*-E-cinnamoyl-β-D-glucopyranoside)Procyanidin B4	AA	Legu	[120]
**286**	3′-galloylprocyanidin B4	AA	Euph	[6]
**287**	Procyanidin B3	AA	Sali	[113,121]
**288**	3-rhamnoside-procyanidin B3	AA	Faga	[122]
**289**	3-glucoside-procyanidin B3	AA	Faga	[122]
**290**	3-*O*-β-D-glucopyranoside, 3′-*O*-(2-*O*-E-cinnamoyl-β-D-glucopyranoside)procyanidin B3	AA	Legu	[120]
**291**	Procyanidin B3 3′-rhamnoside	AA	Faga	[122]
**292**	Procyanidin B3 3′-*O*-glucoside	AA	Rosa	[123]
**293**	Procyanidin B3 7-glucoside	AA	Poly	[124]
**294**	Catechin-(4α→8)-catechin 7-xyloside	AA	Betu	[125]
**295**	3-galloylprocyanidin B3	AA	Rosa	[126]
**296**	3,3′-di-Ac-3′′′-*O*-β-D-glucopyranoside procyanidin B3	AA	Poly	[127]
**297**	3′-*O*-(1-Hydroxy-6-oxo-2-cyclohexene-1-carboxylate)procyanidin B3	AA	Sali	[119]
**298**	Epicatechin-(4β→8)-ent-epicatechin	AA	Arec	[128]
**299**	Ent-epicatechin-(4α→8)-epicatechin	AA	Arec	[128]
**300**	Ent-epicatechin-(4α→8)-catechin	AA	Arec	[128]
**301**	Ent-epicatechin-(4α→8)-ent-epicatechin	AA	Arec	[128]
**302**	3-*O*-(3,4,5-trihydroxybenzoyl)ent-epicatechin-(4α→8)-ent-epicatechin	AA	Malp	[101]
**303**	3′-*O*-(3,4,5-trihydroxybenzoyl)ent-epicatechin-(4α→8)-ent-epicatechin	AA	Malp	[101]
**304**	3,3′-bis-*O*-(3,4,5-trihydroxybenzoyl)ent-epicatechin-(4α→8)-ent-epicatechin	AA	Malp	[101]
**305**	Auricassidin	AA	Legu	[129]
**306**	3,3′,4′,5,7-pentahydroxyflavan-(4→8)-3,4′,5,7-tetrahydroxyflavan	AA	Legu	[130]
**307**	Epicatechin-(4β→8)-epiafzelechin	AA	Legu	[131,132]
**308**	Catechin-(4α→8)-epiafzelechin	AA	Legu	[133]
**309**	Epicatechin-(4β→8)-ent-epiafzelechin	AA	Legu	[134]
**310**	Ent-epicatechin-(4α→8)-epiafzelechin	AA	Legu	[134]
**311**	Ent-epicatechin-(4α→8)-ent-epiafzelechin	AA	Legu	[134]
**312**	Epiguibourtinidol-(4β→8)-epicatechin	AA	Legu	[135]
**313**	Guibourtinidol-(4β→8)-epicatechin	AA	Legu	[105]
**314**	Guibourtinidol-(4β→8)-epiafzelechin	AA	Legu	[105]
**315**	Guibourtinidol-(4α→8)-epicatechin	AA	Legu	[104]
**316**	Guibourtinidol-(4α→8)-epiafzelechin	AA	Legu	[105]
**317**	Guibourtinidol-(4β→8)-catechin	AA	Legu	[104]
**318**	Guibourtinidol-(4α→8)-catechin	AA	Legu	[104]
**319**	Calodenin C	AA	Legu	[136]
**320**	Ent-guibourtinidol-(4β→8)-epicatechin	AA	Legu	[105]
**321**	Epiafzelechin-(4β→8)-epicatechin	AA	Legu, Poly	[134,137]
**322**	3′-*O*-(3,4,5-trihydroxybenzoyl)epiafzelechin-(4β→8)-epicatechin	AA	Poly	[137]
**323**	3,3′-bis-*O*-(3,4,5-trihydroxybenzoyl)epiafzelechin-(4β→8)-epicatechin	AA	Poly	[137]
**324**	Ouratea proanthocyanidin A	AA	Cela, Ochn	[138]
**325**	Ouratea proanthocyanidin B	AA	Cela, Ochn	[138]
**326**	Epiafzelechin-(4ξ→8)-epicatechin	AA	Poly	[94]
**327**	3′-*O*-(4-hydroxybenzoyl)epiafzelechin-(4ξ→8)-epicatechin	AA	Poly	[94]
**328**	3′-*O*-(3-hydroxy-4,5-dimethoxybenzoyl)epiafzelechin-(4ξ→8)-epicatechin	AA	Poly	[94]
**329**	Gambiriin C	AA	Rubi	[139]
**330**	Afzelechin-(4α→8)-epicatechin	AA	Rhiz	[140]
**331**	3′-*O*-(4-hydroxy-3-methoxybenzoyl), 3-*O*-α-L-rhamnopyranoside-afzelechin-(4α→8)-epicatechin	AA	Euph	[141]

* Arec: Arecaceae; Aizo: Aizoaceae; Betu: Betulaceae; Faga: Fagaceae; Hama: Hamamelidaceae; Jugl: Juglandaceae; and Vita: Vitaceae.

**Table 7:** The 4-8′′ subtype of biflavonoids.

No.	Compounds Name	Monomer Type	Origin (Family *)	References
**332**	3′-*O*-(4-hydroxy-3,5-dimethoxybenzoyl), 3-*O*-α-L-rhamnopyranoside-afzelechin-(4α→8)-epicatechin	AA	Euph	[141]
**333**	afzelechin-(4α→8)-catechin	AA	Rhiz, Rosa	[121,140]
**334**	3-*O*-α-L-rhamnopyranoside-afzelechin-(4α→8)-catechin	AA	Faga	[142]
**335**	3-*O*-β-D-glucopyranoside-afzelechin-(4α→8)-catechin	AA	Faga	[142]
**336**	Epiafzelechin-(4β→8)-ent-epicatechin	AA	Legu	[132]
**337**	Ent-epiafzelechin-(4α→8)-epicatechin	AA	Legu	[132]
**338**	Ent-epiafzelechin-(4α→8)-ent-epicatechin	AA	Legu	[134]
**339**	Ichangol	AA	Adox	[143]
**340**	Epicatechin-(4β→8)-epicatechin-(4β→2)-phloroglucinol	AA	Legu, Pina	[109,144]
**341**	Epigallocatechin-(4β→8)-epicatechin-(4β→2)-phloroglucinol	AA	Legu	[109]
**342**	Epigallocatechin-(4β→8)-epigallocatechin-(4β→2)-phloroglucinol 3,3′-digallate	AA	Cist	[145]
**343**	Catechin-(4α→8)-epicatechin-(4β→2)-phloroglucinol	AA	Pina	[144]
**344**	Gallocatechin-(4α→8)-epigallocatechin-(4β→2)-phloroglucinol	AA	Cist	[145]
**345**	Epirobinetinidol-(4β→8)-catechin	AA	Legu	[146]
**346**	Robinetinidol-(4β→8)-epigallocatechin	AA	Mimo	[147]
**347**	Robinetinidol-(4β→8)-epigallocatechin-3′-gallate	AA	Mimo	[147]
**348**	Robinetinidol-(4α→8)-epigallocatechin	AA	Mimo	[147]
**349**	Robinetinidol-(4α→8)-epigallocatechin-3′-gallate	AA	Mimo	[147]
**350**	Robinetinidol-(4β→8)-catechin	AA	Legu	[148]
**351**	Robinetinidol-(4α→8)-gallocatechin	AA	Legu	[149]
**352**	Robinetinidol-(4α→8)-catechin	AA	Legu	[149]
**353**	Prodelphinidin B2	AA	Phyl, Legu, Myri	[100]
**354**	3′-*O*-(4-hydroxybenzoyl)prodelphinidin B2	AA	Legu	[150]
**355**	3-*O*-galloylprodelphinidin B2	AA	Cist, Poly	[99,151]
**356**	3′-galloylprodelphinidin B2	AA	Cist, Myri, Thea	[6,99,100,152]
**357**	Rhodisin	AA	Myri, Cras	[100,153]
**358**	Rhodisinoside	AA	Cras	[153]
**359**	Epicatechin-(4β→8)-epigallocatechin-3-*O*-gallate	AA	Thea	[98]
**360**	Epicatechin-3-*O*-gallate-(4β→8)-epigallocatechin-3-*O*-gallate	AA	Thea	[98]
**361**	Epicatechin-(4β→8)-4′-*O*-methylepigallocatechin	AA	Cela	[154]
**362**	Epigallocatechin-(4β→8)-epicatechin-3-*O*-gallate	AA	Thea	[155]
**363**	Prodelphinidin B1	AA	Cist, Legu	[150,156]
**364**	3-galloylprodelphinidin B1	AA	Cist, Hama	[117,156]
**365**	3,3′-digalloylprodelphinidin B1	AA	Myri	[100]
**366**	Epigallocatechin-(4β→8)-4′-*O*-methylgallocatechin	AA	Legu	[157]
**367**	Epicatechin-(4β→8)-gallocatechin	AA	Phyl	[158]
**368**	Epicatechin-(4β→8)-4′-*O*-methylgallocatechin	AA	Legu	[157]
**369**	Epigallocatechin-(4β→8)-catechin	AA	Legu, Pina	[117,159]
**370**	3′′′-Deoxy, 3-*O*-(3,4,5-trihydroxybenzoyl)epigallocatechin-(4β→8)-catechin	AA	Hama	[117]
**371**	Prodelphinidin B4	AA	Thea, Phyl, Gros	[160]
**372**	Gallocatechin-(4α→8)-epigallocatechin-3-*O*-(4-hydroxybenzoate)	AA	Mimo	[150]
**373**	3′-galloylprodelphinidin B4	AA	Thea	[98]
**374**	4′′,4′′′-di-me ether-prodelphinidin B4	AA	Legu	[150]
**375**	Catechin-(4α→8)-epigallocatechin	AA	Thea	[161]
**376**	Catechin-(4α→8)-epigallocatechin-3-*O*-gallate	AA	Thea	[161]
**377**	Gallocatechin-(4α→8)-epicatechin	AA	Thea	[161]
**378**	Prodelphinidin B3	AA	Faga, Rham	[162,163]
**379**	4′′,4′′′-di-*O*-methylprodelphinidin B3	AA	Legu	[157]
**380**	Catechin-(4α→8)-gallocatechin	AA	Cist	[156]
**381**	Prodelphinidin C	AA	Hama, Myri, Faga, Sali	[117,160,163,164]
**382**	Epifisetinidol-(4β→8)-epicatechin	AA	Legu	[135]
**383**	Epifisetinidol-(4β→8)-catechin	AA	Legu	[165]
**384**	Fisetinidol-(4β→8)-epicatechin	AA	Legu	[166]
**385**	Fisetinidol-(4α→8)-epicatechin	AA	Legu	[166,167]
**386**	Fisetinidol-(4β→8)-catechin	AA	Legu	[166]
**387**	Fisetinidol-(4α→8)-catechin	AA	Legu	[168]
**388**	Fisetidinol-(4α→8)-3-*O*-galloylcatechin	AA	Legu	[166]
**389**	Ent-fisetinidol-(4β→8)-epicatechin	AA	Anac	[149]
**390**	Ent-fisetinidol-(4β→8)-catechin	AA	Anac, Legu	[149,169]
**391**	Ent-fisetinidol-(4α→8)-catechin	AA	Anac, Legu	[149]

* Adox: Adoxaceae; Cela: Celastraceae; Cras: Crassulaceae; Gros: Grossulaceae; Mimo: Mimosaceae; Phyl: Phyllanthaceae; Rhiz: Rhizophoraceae; and Rubi: Rubiaceae.

**Table 8:** The 4′-8′′, 5-5′′, 6-6′′, 6-γ, 6-8′′, 7-7′′, and 8-8′′ subtypes of biflavonoids.

Subtype	No.	Compounds Name	Monomer Type	Origin (Family *)	References
4′-8′′	**392**	5,3′,5′′,4′′′-tetrahydroxy-3′′′,5′′′-dimethoxy-biflavone(4′→8′′)-7-*O*-(2-rhamnoside)rhamnoside	BB	Apia	[170]
5-5′′	**393**	8,8′-bis(7,8-dihydroxy-2-C-methyl-2H-1-benzopyran-5-yl)-4,4′-dimethoxy-[5,5′-bi-6H-furo[3,2-h][1]benzopyran]-6,6′-dione	BB	Legu	[171]
	**394**	3-C-(6-deoxy-α-L-mannopyranosyl)-3′-C-α-d-glucopyranosyl-2,2′-bis(4-hydroxyphenyl)-7,7′-dimethyl-[5,5′-bi-4H-1-benzopyran]-4,4′-dione	BB	Legu	[171]
6-6′′	**395**	Succedaneaflavanone	AA	Anac	[1]
	**396**	6,6′′-bigenkwanin	BB	Ochn	[172]
6-γ	**397**	8-methylsocotrin-3′-methoxy-4′-ol	AG	Drac	[1]
	**398**	8-methylsocotrin-4′-methoxy-3′-ol	AG	Drac	[1]
	**399**	8-methylsocotrin-3-methoxy-4-ol	AG	Drac	[1]
	**400**	8-methylsocotrin-4-methoxy-3-ol	AG	Drac	[1]
	**401**	8-methylsocotrin-4-ol	AG	Drac	[1]
6-8′′	**402**	6,8′′-bigenkwanin	BB	Arau, Ochn	[172,173]
	**403**	Agathisflavone	BB	Anac, Ochn	[173]
	**404**	7,7′′,4′′′-tri-*O*-methylagathisflavone	BB	Arau	[1,174]
	**405**	7,4′′′-di-*O*-methylagathisflavone	BB	Arau	[1]
	**406**	Agathisflavone A	BB	Arau	[173,174]
	**407**	Ouratine A	BB	Ochn	[1,175]
	**408**	Agatisflavone	AB	Ochn	[1]
	**409**	7,4′,7′′,4′′′-tetra-*O*-methylcupressuflavone	AB	Sela	[7]
	**410**	7,4′,7′′-tri-*O*-methylcupressuflavone	AB	Arau	[1]
	**411**	7,7′′-di-*O*-methylcupressuflavone	AB	Arau	[1]
	**412**	Rhusflavone	AB	Anac	[176]
	**413**	Lateriflavanone	AB	Clus	[1]
	**414**	4′′′-*O*-methylagatisflavone	AB	Clus, Ochn	[54,177]
	**415**	Rhusflavanone	AA	Anac	[178]
	**416**	6,8′′-binaringenin	AA	Clus	[178]
	**417**	3′′′,4′,4′′′,5,5′′,7,7′′-hepta-me ether-3,3′′,3′′′,4′,4′′′, 5,5′′,7,7′′-nonahydroxy-6,8′′-biflavanone	AA	Ochn	[179]
	**418**	Ouratine B	BB	Ochn	[175]
	**419**	4′′′-*O*-methylagathisflavone	BB	Ochn	[177]
	**420**	7′′-*O*-methylagathisflavone	BB	Ochn	[180]
	**421**	Agathisflavone B	BB	Arau	[174]
7-7′′	**422**	4′-methoxy-7,7′′-biflavone	BB	Legu	[181]
8-8′′	**423**	Cupressuflavone	AA	Anac, Arau, Cupr	[182]
	**424**	3,3′′-dihydroxycupressuflavone	BB	Thea	[183]
	**425**	4′-*O*-methylcupressuflavone	BB	Clus	[54]
	**426**	Mesuaferrone B	AB	Anac, Clus	[178,184]
	**427**	4′,4′′′-di-*O*-methylcupressuflavanone	AA	Comp	[185]
	**428**	(R)4′-*O*-β-D-glucopyranoside-cupressuflavone	AA	Cupr	[186]
	**429**	(S)4′-*O*-β-D-glucopyranoside-cupressuflavone	AA	Cupr	[186]
	**430**	7-me ether-cupressuflavone	BB	Arau	[173,187]
	**431**	8,8′′-bigenkwanin	BB	Arau, Cupr	[186,187]
	**432**	W11	BB	Arau, Phyl	[188]
	**433**	4′,7,7′′-tri-*O*-methylcupressuflavone	BB	Arau	[187]
	**434**	WB1	BB	Arau	[173,187]
	**435**	Moghatin	BB	Malv	[189]
	**436**	Neorhusflavanone	AA	Anac, Calo	[184,190]

* Apia: Apiaceae; Calo: Calophllaceae; Comp: Compositae; Drac: Dracaenaceae; and Rham: Rhamnaceae.

**Table 9:** The C-linear fragment-C subtypes of biflavonoids.

Subtype	No.	Compounds Name	Monomer Type	Origin (Family *)	References
3′-*O*-3′′′	**437**	Sparinaritin	AB	Chry	[191]
3-*O*-4′′	**438**	Epioritin(4β-3)-epioritin-4β-ol	AA	Legu	[20]
3-*O*-4′′′	**439**	Delicaflavone	BB	Sela	[7]
	**440**	5,7,4′,5′′-tetrahydroxy-7′′-metroxy-[3-*O*-4′′′]-biflavone	BB	Sela	[7]
	**441**	Chrysocauloflavone I	AB	Sela	[7]
	**442**	Chrysocauloflavone II	AB	Sela	[7]
	**443**	Baeckein E	AB	Myrt	[192]
	**444**	Baeckein C	AB	Myrt	[192]
	**445**	Baeckein D	AB	Myrt	[192]
3′-*O*-4′′′	**446**	Ochnaflavone	BB	Ochn	[7,193]
	**447**	Ochnaflvone 7′′-*O*-β-D-glucopyranoside	BB	Ochn	[194]
	**448**	2′′,3′′-dihydroochnaflavone	AB	Ochn	[7,195]
	**449**	2,3-dihydro-4′,7,7′′-tri-*O*-methylochnaflavone	AB	Ochn	[7,196]
	**450**	Sulcatone A	AB	Ochn	[197]
	**451**	4′-me ether-ochnaflavone	BB	Ochn	[193]
	**452**	7-*O*-methylochnaflavone	BB	Ochn	[198]
	**453**	7′′-*O*-methylochnaflavone	BB	Ochn	[199]
	**454**	4′,7-di-*O*-methylochnaflavone	BB	Ochn	[193]
	**455**	2,3-dihydroochnaflavone	AB	Ochn	[200]
	**456**	2,3-dihydro-7-*O*-methylochnaflavone	AB	Ochn	[200]
	**457**	2,3-dihydro-7′′-*O*-methylochnaflavone	AB	Ochn	[9]
	**458**	2′′,3′′-dihydro-7′′-*O*-methylochnaflavone	AB	Ochn	[9]
	**459**	2,2′′,3,3′′-tetrahydroochnaflavone	AA	Para	[201]
	**460**	2,2′′,3,3′′-tetrahydro-7-*O*-methylochnaflavone	AA	Ochn, Para	[201]
	**461**	2,2′′,3,3′′-tetrahydro-7,7′′-di-*O*-methylochnaflavone	AA	Para	[202]
	**462**	3′′′-hydroxyochnaflavone	BB	Rubi	[203]
	**463**	6,6′′-dimethylochnaflavone	BB	Sela	[1]
	**464**	2,3-dihydro-6,6′′-dimethylochnaflavone	AB	Sela	[196]
	**465**	Hypnumbiflavanoid B	AA	Hypn, Ochn	[197,204,205]
3-*O*-7′′	**466**	3-*O*-7′′-biluteolin	BB	Aste	[206]
3′-*O*-7′′	**467**	Lophirone L	BB	Ochn	[52]
	**468**	Lophirone penta-acetate	BB	Ochn	[52]
	**469**	5,7,4′,5′′,3′′′,4′′′′-hexahydroxy-3′′-*O*-β-glucosyl-3′,7′′-*O*-biflavone	BB	Legu	[207]
	**470**	5,7,4′,5′′,3′′′,4′′′′-hexahydroxy-3,3′′-di-*O*-β-glucosyl-3′,7′′-*O*-biflavone	BB	Vita	[208]
4-*O*-4′′	**471**	Epimesquitol(4β-4)-epioritin-4β-ol	AA	Legu	[20]
4′-*O*-4′′′	**472**	Achyrobichalcone	GG	Aste	[209]
	**473**	Loniflavone	BB	Capr	[1]
	**474**	3′-*O*-methylloniflavone	BB	Capr	[210]
	**475**	Oniflavone	BB	Capr	[210]
	**476**	Ericoside	AB	Eric	[211]
4′-*O*-6′′	**477**	Hinokiflavone	BB	Cupr, Psil, Sela, Taxo, Cyca	[212,213,214,215,216]
	**478**	Isocryptomerin	BB	Cupr, Sela, Taxo	[7,213,214,215,217]
	**479**	Neocryptomerin	BB	Podo	[7]
	**480**	Cryptomerin B	BB	Taxo	[7,214,218]
	**481**	Chamaecyparin	BB	Cupr, Sela	[7,217,219]
	**482**	2,3-dihydrohinokiflavone	AB	Cupr, Cyca	[7,215,216]
	**483**	(2S)-2,3-dihydroisocryptomerin	AB	Sela	[7,37]
	**484**	2′′,3′′-dihydrohinokiflavone	AB	Sela	[7,220]
	**485**	2′′,3′′-dihydroisocryptomerin	AB	Sela	[7,221]
	**486**	7-*O*-methyl-2′′,3′′-dihydroisocryptomerin	AB	Sela	[221]
	**487**	Taiwaniaflavone B	AB	Capr	[7,79]
	**488**	(2S,2S’’)-2,3,2′′,3′′-tetrahydrohinokiflavone	AA	Cyca	[7,222]
	**489**	7′′-*O*-methyl-2,3,2′′,3′′-tetrahydrohinokiflavone	AA	Sela	[7]
	**490**	7,4′′-di-*O*-methyl-2,3,2′′,3′′-tetrahydrohinokiflavone	AA	Sela	[7]
	**491**	Oliveriflavone A	AA	Capr	[85]
	**492**	Cryptomerin A	BB	Taxo	[215,218]
	**493**	2,2′′,3,3′′-tetrahydro-7,7′′-di-*O*-methylhinokiflavone	AA	Cyca	[223]
	**494**	2,3-dihydrochamaecyparin	AB	Sela	[39]
4′-*O*-7′′	**495**	Brevipedicelone E	BB	Clus	[224]
4′-*O*-8′′	**496**	Lanaroflavone	BB	Anac, Lana	[225,226]
	**497**	7-*O*-methyllanaroflavone	BB	Ochn	[227]
	**498**	4′′′,7-di-*O*-methyllanaroflavone	BB	Ochn	[227]
	**499**	7,7′′-di-*O*-methyllanaroflavone	BB	Ochn	[228]
5-*O*-5′′	**500**	Potifulgene	AA	Rosa	[1]
6-*O*-7′′	**501**	Masazinoflavanone	AA	Anac	[229]
7-*O*-7′′	**502**	(myricetin-3-*O*-α-L-rhamnoside(C7I-*O*-C7II)myricetin-3-*O*-α-L-rhamnoside	BB	Legu	[230]
6-C-8′′	**503**	Bosistoabiflavanone	AA	Ruta	[231]
8-C-8′′	**504**	Ttrianguletin	BG	Adia	[20]
	**505**	Pentagrametin	AB	Adia	[20]
	**506**	Di(8-catechinyl)methane	AA	Malv	[232]
	**507**	3-*O*-β-D-glucopyranoside-malvidin 8-(8-ethylcatechin)	AC	Red wine	[233]
	**508**	3,3′-bis-*O*-[β-d-xylopyranosyl-(1→2)-[4-hydroxy-3,5-dimethoxy-E-cinnamoyl-(→6)-β-d-glucopyranosyl-(1→6)]-β-d-galactopyranoside]	CC	Apia	[234]
Others	**509**	Carinoside A	BE	Gent	[235]
	**510**	Mesuferrol A	II	Clus	[236]
	**511**	Mesuferrol B	II	Clus	[236]

* Adia: Adiantaceae; Chry: Chrysobalanaceae; Gent: Gentianaceae; Hypn: Hypnaceae; Para: Paracryphiaceae; Lana: Lanariaceae; Psil: Psilotaceae and Ruta: Rutaceae.

**Table 10:** Complex biflavonoids. (Simple-ring type).

Subtype	No.	Compounds Name	Monomer Type	Origin (Family *)	References
C-C & C-C	**512**	Licobichalcone	GG	Legu	[237]
C-C & C-C-C	**513**	Selacyclicbifkavone A	AB	Sela	[7]
C-C & C-*O*-C	**514**	Licoagrodin	AD	Legu	[20]
	**515**	Daphnodorin A	AG	Thym	[29,238]
	**516**	Daphnodorin B	AG	Thym	[29,238]
	**517**	Dihydrodaphnodorin B	AG	Thym	[239]
	**518**	Daphnodorin J	AG	Thym	[29,238,240]
	**519**	Daphnodorin E	AA	Thym	[29]
	**520**	Daphnodorin H	AA	Thym	[29]
	**521**	4′-methoxydaphnodorin E	AA	Thym	[241]
	**522**	Daphnodorin F	AA	Thym	[29]
	**523**	Daphnodorin G	AA	Thym	[29]
	**524**	Lawsonia biflavone A	AB	Lyth	[230]
	**525**	3′-*O*-methyldaphnodorin G	AA	Thym	[242]
	**526**	4′′-*O*-methyldaphnodorin E	AA	Thym	[241]
	**527**	3-*O*-methyldaphnodorin H	AA	Thym	[242]
	**528**	3′-*O*-methyldaphnodorin H	AA	Thym	[242]
	**529**	Daphnogirin A	AA	Thym	[243]
	**530**	Daphnogirin B	AA	Thym	[243]
	**531**	Ephedrannin B	AB	Ephe, Daph	[244,245]
	**532**	Ephedrannin A	AB	Ephe, Daph, Vita	[244,245]
	**533**	Ent-epiafzelechin-(2α→7, 4α→8)-quercetin	AB	Rosa	[246]
	**534**	Proanthocyanidin A5′	AA	Ephe, Rosa	[247,248]
	**535**	3-*O*-α-L-arabinopyranosylproanthocyanidin A5′	AA	Malv	[248]
	**536**	3-*O*-β-D-galactopyranosylproanthocyanidin A5′	AA	Malv	[248]
	**537**	Pavetannin A2	AA	Ephe, Rosa, Rubi	[249]
	**538**	3-*O*-α-L-arabinopyranosylpavetannin A2	AA	Malv	[232]
	**539**	Ent-epicatechin-(2α→7,4α→8)-ent-catechin	AA	Rubi	[250]
	**540**	Ent-epicatechin-(2α→7,4α→8)-ent-epicatechin	AA	Ephe	[248]
	**541**	Proanthocyanidin A2	AA	Sapi, Legu, Laur	[251,252,253]
	**542**	3′-*O*-trans-cinnamoylproanthocyanidin A2	AA	Legu	[254]
	**543**	Proanthocyanidin A1	AA	Sapi, Legu, Laur	[253]
	**544**	Proanthocyanidin A4	AA	Sapi, Rubi	[250]
	**545**	Baeckein F	AB	Myrt	[1]
	**546**	Baeckein G	AB	Myrt	[255]
	**547**	Baeckein H	AB	Myrt	[1]
	**548**	Baeckein I	AB	Myrt	[255]
	**549**	Lophirone C	GG	Ochn	[256,257]
	**550**	Dihydrolophirone C	GG	Ochn	[257]
	**551**	Isolophirone C	GG	Ochn	[257]
	**552**	Lophirone K	GG	Ochn	[258]
	**553**	Lophirone F	GG	Ochn	[259]
	**554**	Lophirone G	GG	Ochn	[259]
	**555**	Lophirone L‡	GG	Ochn	[260]
C-*O*-C & C-*O*-C	**556**	Dysoverine D	AB	Berb	[261]
	**557**	Dysoverine F	AB	Berb	[261]
	**558**	Dysoverine A	AB	Berb	[261]
	**559**	(2R,2′R,3S,3′S,4α,4′α)-3′,4′,7-trihydroxyflavan-(3→*O*→4)(4→*O*→3)-3′,4′,7-trihydroxyflavan	AA	Legu	[262]

* Berb: Berberidaceae; Daph: Daphniphyllaceae; Ephe: Ephedraceae; Laur: Lauraceae; and Lyth: Lythraceae.

**Table 11:** The other types of bioflavonoids.

Subtype	No.	Compounds Name	Monomer Type	Origin (Family *)	References
Bicyclic type	**560**	Daphnodorin M	AG	Thym	[29]
	**561**	Daphnodorin N	AG	Thym	[29]
	**562**	Stelleranol	AG	Thym	[1]
	**563**	Genkwanol B	AG	Thym	[1]
	**564**	Genkwanol C	AG	Thym	[1]
Atom-shared type	**565**	Chamaechromone	EG	Thym	[63]
	**566**	Mohsenone	EG	Thym	[63]
	**567**	Isomohsenone	EG	Thym	[63]
	**568**	Lophirone A	EG	Ochn	[263]
	**569**	Calodenone	EG	Ochn, Anac	[264]
	**570**	Afzelone D	EG	Ochn	[265]
	**571**	Campylopusaurone	AH	Clus	[266]
	**572**	Preussianone	AB	Clus	[267]
	**573**	Paucinervin K	AE	Clus	[268]
	**574**	Lancedatin A	BD	Legu	[20]
	**575**	Lancedatin B	BD	Legu	[20]
Spirobiflavonoids	**576**	Absienol A	AA	Mora	[60]
	**577**	Absienol B	AA	Pina	[1]
	**578**	Absienol C	AA	Mora	[60]
	**579**	Absienol D	AA	Pina	[1]
	**580**	Absienol E	AA	Mora	[60]
	**581**	Absienol F	AA	Pina	[1]
	**582**	Daphnodorin C	AA	Thym	[29]
	**583**	Daphnodorin I	AA	Thym	[29]
	**584**	Genkwanol A	AA	Thym	[269]
	**585**	2′′-hydroxygenkwanol A	AA	Thym	[1]
	**586**	4′-methylgenkwanol A	AA	Thym	[1]
	**587**	Olgensisinol A	AA	Pina	[270]
	**588**	Olgensisinol B	AA	Pina	[270]
	**589**	Olgensisinol C	AA	Pina	[270]
	**590**	Olgensisinol D	AA	Pina	[270]
	**591**	Vitisinol	AA	Pina	[270]
	**592**	Larixinol	AA	Pina	[271]

**Table 12:** The pharmacokinetics of biflavonoids.

Name	Testline	Delivery Route	Doses(mg/kg)	Method	T_1/2_(h)	References
Amentoflavone	Rat plasma	i.p.	10	LC-MS/MS	3.42 ± 1.45	[320]
Amentoflavone	Rat plasma	i.v.	10	LC-MS/MS	5.88 ± 1.78	[320]
Amentoflavone	Rat plasma	p.o.	300	LC-MS/MS	11.3 ± 3.61	[320]
Amentoflavone	Rat plasma	p.o.	4.31	UFLC-MS/MS	2.60 ± 1.34	[322]
Hinokiflavone	Rat plasma	p.o.	4.30	UFLC-MS/MS	2.11 ± 0.29	[322]
Hinokiflavone	Rat plasma	i.v.	1.0	LC-MS/MS	6.10 ± 1.86	[321]

## Data Availability

The data presented in this study are available on request from the corresponding author.
